# Getting ‘ϕψχal’ with proteins: minimum message length inference of joint distributions of backbone and sidechain dihedral angles

**DOI:** 10.1093/bioinformatics/btad251

**Published:** 2023-06-30

**Authors:** Piyumi R Amarasinghe, Lloyd Allison, Peter J Stuckey, Maria Garcia de la Banda, Arthur M Lesk, Arun S Konagurthu

**Affiliations:** Department of Data Science and Artificial Intelligence, Faculty of Information Technology, Monash University, Clayton, VIC 3800, Australia; Department of Data Science and Artificial Intelligence, Faculty of Information Technology, Monash University, Clayton, VIC 3800, Australia; Department of Data Science and Artificial Intelligence, Faculty of Information Technology, Monash University, Clayton, VIC 3800, Australia; OPTIMA ARC Industrial Training and Transformation Centre, Carlton, VIC 3053, Australia; Department of Data Science and Artificial Intelligence, Faculty of Information Technology, Monash University, Clayton, VIC 3800, Australia; OPTIMA ARC Industrial Training and Transformation Centre, Carlton, VIC 3053, Australia; Department of Biochemistry and Molecular Biology, Pennsylvania State University, University Park, PA 16802, United States; Department of Data Science and Artificial Intelligence, Faculty of Information Technology, Monash University, Clayton, VIC 3800, Australia

## Abstract

The tendency of an amino acid to adopt certain configurations in folded proteins is treated here as a statistical estimation problem. We model the joint distribution of the observed mainchain and sidechain dihedral angles ($\langle \phi ,\psi ,\chi_{1},\chi_{2},\ldots \rangle$) of any amino acid by a mixture of a product of von Mises probability distributions. This mixture model maps any vector of dihedral angles to a point on a multi-dimensional torus. The continuous space it uses to specify the dihedral angles provides an alternative to the commonly used rotamer libraries. These rotamer libraries discretize the space of dihedral angles into coarse angular bins, and cluster combinations of sidechain dihedral angles ($\langle \chi_{1},\chi_{2},\ldots \rangle$) as a function of backbone 〈ϕ,ψ〉 conformations. A ‘good’ model is one that is both concise and explains (compresses) observed data. Competing models can be compared directly and in particular our model is shown to outperform the Dunbrack rotamer library in terms of model complexity (by three orders of magnitude) and its fidelity (on average 20% more compression) when losslessly explaining the observed dihedral angle data across experimental resolutions of structures. Our method is unsupervised (with parameters estimated automatically) and uses information theory to determine the optimal complexity of the statistical model, thus avoiding under/over-fitting, a common pitfall in model selection problems. Our models are computationally inexpensive to sample from and are geared to support a number of downstream studies, ranging from experimental structure refinement, *de novo* protein design, and protein structure prediction. We call our collection of mixture models as PhiSiCal (ϕψχal).

**Availability and implementation:**

PhiSiCal mixture models and programs to sample from them are available for download at http://lcb.infotech.monash.edu.au/phisical.

## 1 Introduction

The 20 naturally occurring amino acids form the nature’s part list from which proteins are made within the cells of organisms. In all amino acids a central carbon atom (the *α*-carbon) binds an amino group (-NH_2_), a carboxylic acid (-COOH) group, and a hydrogen atom, but differ in the fourth group attached, a sidechain (R).

Protein polypeptide chains of amino acids fold into compact three-dimensional shapes stabilized by inter-atomic interactions between the amino acids. The resultant amino acid conformations are determined by the varying degrees of rotations (‘torsions’) around the atomic bonds, subject to the physics and chemistry of protein folding.

**Figure 1.**
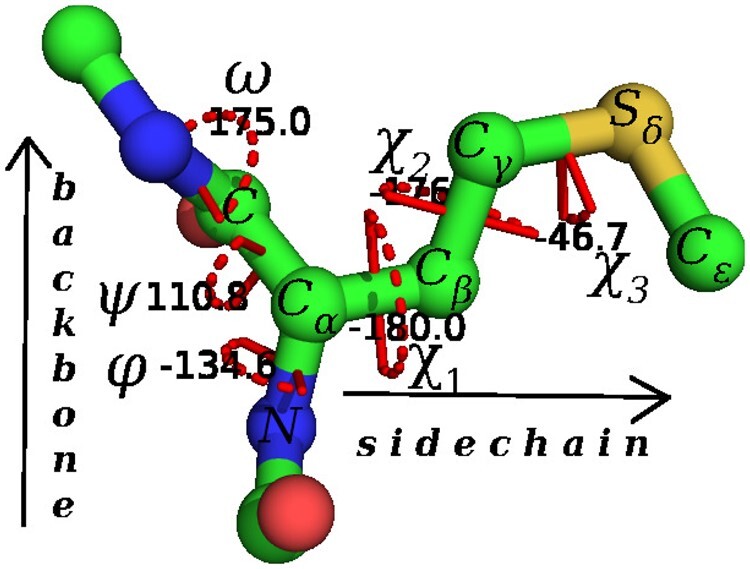
The amino acid Methionine (MET) has its conformation specified by six dihedral angles: $\langle \phi ,\psi ,\omega ,\chi_{1},\chi_{2},\chi_{3}\rangle$, where each angle is in the range $\left(-180^{{}^{\circ}},180^{{}^{\circ}}\right]$. (The angles shown above are those observed for MET67 in the fibroblast growth factor protein, 1BAR. Note that the value of $\chi_{1}=-180^{{}^{\circ}}$ for the C${}_{\alpha }$–C${}_{\beta }$ bond corresponds to the *trans* conformation.) For MET, the sidechain, or R group, is -C${}_{\beta }$-C${}_{\gamma }$-S${}_{\delta }$-C${}_{\epsilon }$.

Any torsion can be mathematically calculated as a ‘dihedral angle’*—*the angle between two planes—defined by four points (here, the coordinates of successively bonded atoms) sharing a common basis vector (here, the central bond around which the torsion is being measured) (IUPAC-IUB Commission, 1970). Thus, any amino acid conformation can be described as a vector of dihedral angles, conventionally denoted by the sequence of symbols, $\langle \phi ,\psi ,\omega ,\chi_{1},\chi_{2},\ldots \rangle$ (see Fig. 1).

Across all amino acids, the symbols 〈ϕ,ψ,ω〉 are used to denote the dihedral angles around the backbone bonds, whereas $\langle \chi_{1},\chi_{2},\ldots \rangle$ are used to denote exclusively the torsions around the sidechain bonds. Note that the number of sidechain dihedral angles depends on the sidechain (R) groups, and hence varies with the amino acid type.

Analysis of the observed distributions of backbone and sidechain dihedral angles has been an object of intense interest since the early protein structural and biophysical studies: Ramachandran et al. (1963), Janin and Wodak (1978), McGregor et al. (1987), Dunbrack and Karplus (1993), Dunbrack and Cohen (1997), Dunbrack (2002), and Shapovalov and Dunbrack (2007, 2011). This interest is fuelled by the need for accurate statistical models that can effectively characterize the observed dihedral angle distributions of proteins, as these models are used by techniques for protein experimental structure determination, computational prediction, rational design, and many other protein structural analyses.

One of the results has been the creation of rotamer libraries. A ‘rotamer’ is any rotational preference of the set of dihedral angles along the sidechain bonds within amino acids. These libraries are compiled from the statistical clustering of sidechain conformations of known protein structures (Dunbrack 2002). Rotamer libraries are 2-fold: backbone independent and backbone dependent. Backbone-dependent rotamer libraries contain rotameric preferences conditioned on any observed backbone dihedral angles (Dunbrack and Karplus 1993; Dunbrack and Cohen 1997; Shapovalov and Dunbrack 2011), and differ from the backbone-independent libraries which simply cluster sidechain conformations agnostic to the backbone conformation of amino acids (Ponder and Richards 1987; Lovell et al. 2000).

Rotamer libraries derive sidechain conformation statistics using coarse quantization of the observed rotation space for each sidechain dihedral angle. This discretization often uses an angular interval of $120^{{}^{\circ}}$ regions, yielding a $\left(-60^{{}^{\circ}},60^{{}^{\circ}},180^{{}^{\circ}}\right)$ trisection of the rotational space, that corresponds to the staggered conformation of two *sp*^3^-hybridized atoms (Dunbrack 2002). Under such a discretization, each rotamer clusters around a mean conformational preference over a discretized interval. Such rotameric descriptions of sidechain torsions have the advantage of yielding a computationally tractable conformation space when inferring rotational preferences of individual amino acids and fitting them in several protein modelling tasks [e.g. in *de novo* protein design (Desmet et al. 1992)].

However, such discretizations can also bias downstream studies, e.g. leading to inaccurate modelling of the details of inter-atomic interactions for protein docking (Wang et al. 2005), and to imprecise protein conformational energy landscapes (Grigoryan et al. 2007), among others (Lassila 2010). Further, several of the outermost dihedral angles of certain amino acids – *χ*_3_ of glutamic acid (GLU) and glutamine (GLN), *χ*_2_ of aspartic acid (ASP), and asparagine (ASN) – flout the three-way discretization of its rotational space and hence lead to broad and visually featureless distributions that have resisted attempts to characterize the observed spread accurately (Lovell et al. 1999; Shapovalov and Dunbrack 2011). As discussed by Schrauber et al. (1993), in these instances the rotameric representation of sidechain conformations is limited and large deviations of *χ* angles from the canonical values can be observed. The existence of such ‘non-rotameric’ conformations was also discussed in detail by Heringa and Argos (1999).

An approach employed to mitigate this issue is to calculate distribution frequencies on a finer grid (Schrauber et al. 1993). A more accurate approach is to model the distribution over a continuous space, as this would result in a finer representation minimizing information loss. This is the approach taken by BASILISK (Harder et al. 2010) which formulates a probabilistic model that represents the torsion angles in a continuous space. However, it uses a single probabilistic model for all the amino acids.

The Dunbrack rotamer library (Dunbrack and Karplus 1993; Dunbrack and Cohen 1997; Dunbrack 2002; Shapovalov and Dunbrack 2007, 2011) is a continually maintained and improved rotamer library. It defines the state of the art and is among the most widely used rotamer libraries across many downstream applications that employ them. While this library is backbone dependent, it uses the same supervised-discretized choices. This discretization renders their resultant models both overly complex as well as inaccurate in capturing the observed distributions of dihedral angles when sampled from its libraries (see Section 3).

In this work, we take a different approach by modelling the joint distributions of the observed mainchain and sidechain dihedral angles of individual amino acids by a mixture of a product of von Mises probability distributions. To infer these mixture models, we use the Bayesian and information-theoretic criterion of minimum message length (MML) (Wallace and Boulton 1968; Wallace and Freeman 1987; Wallace 2005). In the theory of learning and generalization, this unsupervised model selection framework falls under the class of statistical inductive inference (Wallace 2005). Among other notable and well-established statistical properties, MML allows an objective trade-off between model complexity and fit—these form two opposing criteria that all model selection problems contend with, but for which MML provides an intuitive, objective, and rigorous reconciliation.

We compared our mixture models inferred for each amino acid with the Dunbrack rotamer library on large datasets containing structures that are non-redundant in sequence and filtered based on high-resolution, B-factor, and R-factor cut-offs. Our results clearly demonstrate that the mixture models we infer outperform the Dunbrack rotamer library both in its model complexity (by three orders of magnitude) and its fidelity (yielding on average 20% more lossless compression) when explaining the observed dihedral angle data. Our MML mixture model library, termed ‘ϕψχal’ supports fast sampling of joint and conditionally distributed dihedral angle vectors to support their use in many downstream studies involving protein structures.

## 2 Methods

### 2.1 Mixture model overview

We present a systematic method of ‘unsupervised’ estimation of a statistical model that can effectively explain any given observations of ‘vectors’ (of any dimension) of dihedral angles using the statistical inductive inference framework of MML (Wallace and Boulton 1968; Wallace 2005; Allison 2018).

Specifically, this work infers a ‘mixture model’ under the Bayesian and information-theoretic criterion of MML, where each component of the mixture defines a ‘product’ of a series of von Mises distributions (Mardia et al. 2000), one for each dihedral angle observed in the specified amino acid. We note that the number of components, their probabilities, and corresponding parameters are all unknown and are inferred unsupervised by our method.

Formally, for a specified amino acid ‘aa’ (i.e. any of the 20 naturally occurring amino acids in proteins), $X=\{x_{1},x_{2},\ldots ,x_{N}\}$ represents an input set of *N* observations of the conformational states of that amino acid. Each $x_{i}\in X$ defines a vector of the *d* dihedral angles (whose terms are specified in some canonical order) as observed in the *i-*th instance of ‘aa’. For example, each instance of the amino acid methionine (see Fig. 1) is defined by a *d *=* *6-dimensional vector containing its dihedral angles $\langle \phi ,\psi ,\omega ,\chi_{1},\chi_{2},\chi_{3}\rangle$. In this case, *X* captures the set of observed instances of various conformational states of methionine derived from a non-redundant set of experimental coordinates in the world-wide protein data bank (Berman et al. 2000).

A ‘mixture model’ is any convex combination of ‘component’ probability density functions used to explain some observed data containing a number of subpopulations (often unknown in advance) within an overall population (Figueiredo and Jain 2002; McLachlan et al. 2019). Specifically, in this work, we consider a mixture model that takes the general form:


(1) $$M(\Lambda )=\sum_{j=1}^{\left|M\right|}w_{j}f(\Theta_{j})\;\text{such that }\sum_{j=1}^{\left|M\right|}w_{j}=1.$$

This defines a continuous probability distribution for a *d*-dimensional random vector $x_{i}=\langle x_{i_{1}},\;x_{i_{2}},\;\ldots ,\;x_{i_{d}}\rangle$ such that $x_{i_{p}}\in (-\pi ,\pi ],\forall 1\leq p\leq d$. Thus, the support for *x_i_* defines a surface of a *d*-Torus (denoted as $T^{d}$). $|M|\in Z^{+}$ denotes the size of the mixture model given by the number of ‘components’ it defines. Each component function $f(\Theta_{j})$ denotes the joint probability distribution of the random vector $x_{i}\in T^{d}$. In this work, each mixture component takes the form of a product of *d* von Mises circular distributions, $f(\Theta_{j})\propto \prod_{p=1}^{d}\;\text{exp}\;(\kappa_{j_{p}}\;\text{cos}({x_{i_{p}}}_{}-\mu_{j_{p}})),$ where each $\langle \mu_{j_{p}},\kappa_{j_{p}}\rangle$ represent the 〈mean,concentration〉 parameters of each von Mises term in the product and $\Theta_{j}={\left\{\langle \mu_{j_{p}},\kappa_{j_{p}}\rangle \right\}}_{\forall 1\leq p\leq d}$ denotes the collection of all von Mises’ parameters of the *j-*th mixture component. Each *w_j_* denotes a mixture components’ respective ‘weight’ which, over all |M| terms in the mixture, add up to 1. Finally, we use Λ as a shorthand to collectively denote all mixture model’s parameters:

1. the ‘number’ of mixture components |M|,
2. the set of ‘weights’ of mixture components ${\left\{w_{j}\right\}}_{\forall 1\leq j\leq |M|}$, and
3. the set of all parameters defining the mixture ‘components’ ${\left\{\Theta_{j}\right\}}_{\forall 1\leq j\leq |M|}\equiv {\left\{{\left\{\langle \mu_{j_{p}},\kappa_{j_{p}}\rangle \right\}}_{\forall 1\leq p\leq d}\right\}}_{\forall 1\leq j\leq |M|}$.

Thus, for any specified amino acid ‘aa’ with its given set of dihedral angle tuples *X*, the goal of this work is to infer a mixture model M that best explains all the observations in *X*. The key challenge in doing so is to estimate the mixture parameters Λ unsupervised. To address this unsupervised estimation problem, we employ the Bayesian and information-theoretic criterion of MML, as follows.

### 2.2 MML inference foundations

#### 2.2.1 MML and model selection

MML is a Bayesian method for hypothesis/model selection. In general terms, if *X* is some given data and *M* is some statistical model describing that data, the joint probability of the model *M* and data *X* is given by the product rule of probability: Pr(M,X)=Pr(M)Pr(X|M). This can be recast in terms of Shannon information based on the observation that the optimal code length to represent any event *E* (with a probability Pr(E)) is given by the measure of Shannon information content quantified (say in bits of information) as $I(E)=-{\;\text{log}\;}_{2}(\mathrm{Pr}(E))$ (Shannon 1948). Expressing the above product rule of probability in terms of Shannon information content, we get:


(2) $$\underset{\text{Total Message Length}}{\underset{︸}{I(M,X)}}=\underset{\text{first part}}{\underset{︸}{I(M)}}+\underset{\text{second part}}{\underset{︸}{I(X|M)}}.$$

In the above equation, the amount of information required to losslessly explain the observed data *X* with a hypothesis/model *M* can be seen as the length of a two-part message: the ‘first part’ contains the information required to state the model *M* losslessly (quantifying the model’s descriptive ‘complexity’), whereas the ‘second part’ contains the information required to state the data *X* ‘given’ the model *M* (quantifying the model’s ‘fit’ with the data). It is easy to see that, in this information-theoretic view, the best model $M^{*}$ is the one whose total two-part message is minimum (optimally trading-off the model’s complexity and fit): $M^{*}=\arg\;\min_{\forall M}\;I(M,X)$. This is equivalent to maximizing the joint probability $\arg\;\max_{\forall M}\mathrm{Pr}(M,X)$. Thus, under the MML framework, any pair of competing models explaining the same data can be compared based on their respective total lengths: the difference in total message lengths derived using any two models gives their log-odds posterior ratio, making this method of model selection Bayesian (Wallace 2005; Allison 2018).

#### 2.2.2 Wallace–Freeman method of parameter estimation using MML

Let M(α) denote a twice-differentiable statistical model with a parameter vector *α* (with |α| number of free parameters) and *X* denote some observed data (containing |X| number of observations). Wallace and Freeman (1987) showed that the total message length of any general model *M* with a vector of parameters *α* can be approximated as where h(α) is the prior probability density of the parameters *α*, det(F(α)) is the determinant of the ‘expected’ Fisher information matrix, L(α) is the negative log-likelihood function of *X* given *α*, $q_{\left|\alpha \right|}$ represents the Conway–Sloane (Conway and Sloane 1984) lattice quantization constant in |α|-dimensional space, and *ϵ* is the uncertainty of each datum in the set *X* of size |X|. Refer to Wallace (2005) and Allison (2018) for details of this method of estimation.


(3) $$\begin{array}{c} I(M(\alpha ),X)\approx \underset{\text{First part}:\;I(M(\alpha ))}{\underset{︸}{\;\text{log}\;\left(\frac{\sqrt{\text{det}(F(\alpha ))}\sqrt[{\frac{2}{\left|\alpha \right|}}]{q_{\left|\alpha \right|}}}{h(\alpha )}\right)}}\\ +\underset{\text{Second part:}I(X|M(\alpha ))}{\underset{︸}{L(\alpha )-|X||\alpha |\;\text{log}(\epsilon )+\frac{\left|\alpha \right|}{2}}}, \end{array}$$

This Wallace and Freeman (1987) method informs the computation of various message length terms in the work presented here.

### 2.3 Message length of a mixture model

Applying the general MML framework to the mixture models introduced in Section 2.1 allows us to characterize the length of the message needed to explain jointly any observed set of dihedral angle vectors *X* using a mixture model M with parameter vector Λ analogously to Equation (2) as


(4) I(M(Λ),X)=I(M(Λ))+I(X|M(Λ)).

This in turn is used to define the objective function we use to estimate an optimal set of mixture model parameters that can losslessly explain itself (M(Λ)) and the observations *X* in the most succinct way in terms of Shannon information: $\Lambda_{\text{MML}}=\arg\;\min_{\forall \Lambda }I(M(\Lambda ),X)$.

#### *2.3.1 Computing* I(M(Λ))*term of Equation (4)*

As described in Section 2.1, Λ denotes the combined set of mixture model parameters $\left(|M|,{\left\{w_{j}\right\}}_{\forall 1\leq j\leq |M|},{\left\{\Theta_{j}\right\}}_{\forall 1\leq j\leq |M|}\right)$. Thus, the Shannon information content in a mixture model can be expressed as the summation of the message lengths terms required to state all its parameters losslessly:


(5) $$I(M(\Lambda ))=\underset{\text{term}\;1}{\underset{︸}{I(|M|)}}+\underset{\text{term}\;2}{\underset{︸}{\sum_{j=1}^{\left|M\right|}I(w_{j})}}+\underset{\text{term}\;3}{\underset{︸}{\sum_{j=1}^{\left|M\right|}I(\Theta_{j})}}.$$

Computation of each of the message length terms on the right-hand side of Equation (5) is described below.

##### Computation of Term 1 of Equation (5)

$|M|\in Z^{+}$ is a countable positive integer and thus can be stated using an universal prior for integers over a variable-length integer code (Allison et al. 2019). We employ the Wallace Tree Code (Wallace and Patrick 1993; Allison et al. 2019) to compute I(|M|) in Equation (5).

##### Computation of Term 2 of Equation (5)

The set of *L*_1_ normalized weight vector ${\left\{w_{j}\right\}}_{\forall 1\leq j\leq |M|}$ can be viewed as a parameter of a multinomial distribution, whose support defines a unit (|M|−1) simplex (Wallace 2005; Allison 2018). Using the Wallace–Freeman method of estimation described in Section 2.2.2, assuming a uniform prior for the weights as a point in a unit (|M|−1) simplex, i.e. the prior $h=(|M|-1)!/\sqrt{\left|M\right|}$, and computing the determinant of the Fisher information matrix for a multinomial distribution (with parameters $\left\{w_{j}\right\}$) as $N^{|M|-1}/\Pi_{j=1}^{\left|M\right|}w_{j}$, it can be shown [as per the first part of Equation (3)] that the message length of Term 2 is given by (Allison 2018):


$$\begin{array}{c} \sum_{j=1}^{\left|M\right|}I(w_{j})=\frac{\left(|M|-1\right)}{2}\text{log}(q_{\left(|M|-1\right)})-\text{log}\;\left(\frac{(|M|-1)!}{\sqrt{\left|M\right|}}\right)\\ +\frac{\left(|M|-1\right)}{2}\text{log}(N)-\frac{1}{2}\sum_{j=1}^{\left|M\right|}\;\text{log}\;(w_{j}) \end{array}.$$

##### Computation of Term 3 of Equation (5)

Recall (from Section 2.1) that each $\Theta_{j}={\left\{\langle \mu_{j_{p}},\kappa_{j_{p}}\rangle \right\}}_{\forall 1\leq p\leq d}$. Thus, $I(\Theta_{j})=\sum_{p=1}^{d}I(\langle \mu_{j_{p}},\kappa_{j_{p}}\rangle \})$. Each $I(\langle \mu_{j_{p}},\kappa_{j_{p}}\rangle \})$ term in the summation is estimated by again applying the Wallace–Freeman method (Section 2.2.2), this time for a von Mises circular distribution. A von Mises distribution defines a probability distribution of a random variable *x* on a circle (i.e. x∈(−π,π]) as a function of its two free parameters, mean μ∈(−π,π] and concentration κ>0: $f(x;\langle \mu ,\kappa \rangle )=\frac{{\;\text{exp}\;}^{\kappa \;\text{cos}(x-\mu )}}{2\pi B_{0}(\kappa )},$ where the denominator on the right-hand side gives the normalization constant of the distribution in terms of the modified Bessel function (of order 0), denoted here as $B_{0}(\kappa )$. More commonly, modified Bessel functions of order *r* are denoted as $I_{r}(\cdot )$. We use *B_r_* here only to avoid confusion with the Shannon information content notation, I(·).

In applying the Wallace–Freeman method, the assumed priors for the two parameters are [as per Kasarapu and Allison (2015)]: $h(\mu )=\frac{1}{2\pi }$ and $h(\kappa )=\frac{\kappa }{{\left(1+\kappa^{2}\right)}^{\frac{3}{2}}}$. Thus, h(〈μ,κ〉)=h(μ)h(κ). We note that the rationale and behaviour of these priors for von Mises has been previously studied (Wallace 2005). The chosen prior on *μ* is uniform (and hence uninformative/flat), giving only general information about the variable being estimated, which makes it suitable. On the other hand, no truly uninformative prior exists for *κ*. The chosen prior ensures the function is smooth (without singularities) and commonly preferred when the data concentration is expected to arise from physical interactions (Wallace 2005).

Further, for some *N* observations of circular angles in the range (−π,π] defined by (say) the set $X=\{x_{1},x_{2},\ldots ,x_{N}\}$, it can be shown that the ‘determinant’ of the expected Fisher information matrix for a von Mises distribution can be characterized as $\text{det}(F(\langle \mu ,\kappa \rangle ))=\kappa NA(\kappa )A^{\prime }(\kappa )$, where $A(\kappa )=\frac{B_{1}(\kappa )}{B_{0}(\kappa )}$ and $A^{\prime }(\kappa )=\frac{d}{d\kappa }A(\kappa )$. Using this prior and determinant, the message length term to state the pair of 〈μ,κ〉 parameters of any single von Mises circular distribution [as per the first part of Equation (3)] can be written as


(6) $$I(\langle \mu ,\kappa \rangle )=\text{log}(q_{2})-\text{log}(h(\langle \mu ,\kappa \rangle ))+\frac{1}{2}\text{log}(\text{det}(F(\langle \mu ,\kappa \rangle ))).$$

#### 2.3.2 Computing I(X|M(Λ)) term of Equation (4)

The second part of Equation (4) deals with explaining the observations of the vectors of dihedral angles *X* using the mixture model parameters that have been stated losslessly via the first part (Section 2.3.1). Using the relationship between Shannon information and probability (Section 2.1), that is, I(·)=−log(Pr(·)), I(X|M(Λ)) can be decomposed using the likelihood of each *d*-dimensional dihedral angle $x_{i_{p}}\in x_{i}\in X$ (assuming independent and identically distributed datum) using the mixture model parameters as where *ϵ* in the above expression denotes the degree of uncertainty of each dihedral angle $x_{i_{p}}$ to estimate its component likelihood over a von Mises distribution. This work sets ϵ=0.0873 radians, based on the observation that the effective precision of 3D atomic coordinate is not better than 0.1Å (Konagurthu et al. 2014).


$$I(X|M(\Lambda ))=\sum_{i=1}^{N}-\text{log}\;\left(\sum_{j=1}^{\left|M\right|}\left(w_{j}\Pi_{p=1}^{d}f\left(x_{i_{p}}|\left\langle \mu_{j_{p}},\kappa_{j_{p}}\right\rangle \right)\epsilon^{d}\right)\right),$$

### 2.4 Search for optimal mixture model parameters

#### 2.4.1 Expectation–maximization (EM)

To search for an optimal mixture model $M(\Lambda_{\text{MML}})$ that minimizes Equation (4), we employ a deterministic EM algorithm commonly employed for statistical parameter estimation problems (Dempster et al. 1977; McLachlan and Basford 1988; McLachlan et al. 2019). EM is an iterative algorithm which, in each iteration, explores local updates to the current parameter estimates to be able to generate new parameter estimates that yield progressively shorter message lengths [in this work, the evaluation of Equation (4)] until convergence.

Let Λ(t) denote the state of the mixture parameters at an iteration indexed by t≥0. Then at each iteration indexed as {1,2,…,t,t+1,…} the EM performs an *E(xpectation)*-step followed by a *M(aximization)*-step, as described below.

##### E-step

Using the current state of parameter estimates after iteration *t*, i.e. Λ(t), the E-step calculates the (probabilistic) ‘responsibilities’ $r_{ij}(t+1)\forall 1\leq i\leq N,1\leq j\leq |M|$ in the next iteration *t *+* *1 as


(7) $$r_{ij}(t+1)=\frac{w_{j}(t)f(x_{i}|\Theta_{j}(t))}{\sum_{j\prime =1}^{\left|M\right|}w_{j\prime }(t)f(x_{i}|\Theta_{j\prime }(t))}.$$

Formally responsibility *r_ij_* is the posterior probability that *x_i_* belonging to *j* and it quantifies the degree to which a component *j* ‘explains’ the data point *x_i_* (McLachlan et al. 2019). From these responsibilities, given *N* observations of dihedral angles, any *j-*th component’s membership in iteration *t *+* *1 is calculated as


$$n_{j}(t+1)=\sum_{i=1}^{N}r_{ij}(t+1)\;\text{and}\;\sum_{j=1}^{\left|M\right|}n_{j}(t+1)=N.$$

##### M-step

In the M-step, the mixture parameters are updated as follows. The set of weights for *t *+* *1 are derived as the MML estimates of parameters of a multistate distribution (Allison 2018) with *N* observations over |M| distinct states while treating ${\left\{n_{j}(t+1)\right\}}_{\forall 1\leq j\leq |M|}$ as each component/state’s number of observed instances (out of *N*):


(8) $$w_{j}(t+1)=\frac{n_{j}(t+1)+\frac{1}{2}}{N+\frac{\left|M\right|}{2}}.$$

Further, the update to each mean parameter of a von Mises distribution (∀1≤j≤|M|,1≤p≤d) is given by where $R_{j_{p}}$ is the ‘vector sum’ of each $x_{i_{p}}$th dihedral angle in the tuple $x_{i}\in X$, weighted by its corresponding responsibility $r_{ij}(t+1)$. We note that this vector sum arises because each dihedral angle is written as a 2D trigonometric coordinate $\left(\text{cos}\;x_{i_{p}},\;\text{sin}\;x_{i_{p}}\right)$ on a unit circle. $\left\|R_{j_{p}}\right\|$ is the vector norm of the resultant vector $R_{j_{p}}$.


(9) $$\mu_{j_{p}}(t+1)=\frac{R_{j_{p}}}{\left\|R_{j_{p}}\right\|},$$

Finally, the update to the concentration parameter $\kappa_{j_{p}}$ of von Mises distribution (∀1≤j≤|M|,1≤p≤d) follows a numerical approach, as solving for the roots of $\frac{\partial }{\partial \kappa }I(\langle \mu ,\kappa \rangle ,X_{p})=0$ has no closed form (see Supplementary Section S1).

#### 2.4.2 Search for the optimal number of mixture components, |M|

A priori, the number of mixture components |M| is unknown, along with other mixture parameters. Thus, the EM algorithm starts with a single component mixture model at iteration *t *=* *0 (i.e. |M|=1). It then follows similar mechanics to that described by Kasarapu and Allison (2015), albeit with some improvements.

Starting from a single-component mixture at t=0, during each iteration (t+1), a set of perturbations, Split, Merge, and Delete are systematically executed on each component of the mixture model Λ(t). We note that each Split of a component increases the number of components |M| by +1, whereas Merge and Delete decrease it by −1. After each such perturbation, the parameters of the resulting new mixture (with increased/decreased number of components) are reestimated using EM updates described in Section 2.4.1 starting with initial parameters assigned deterministically at the E-step. After systematically exploring all of the above perturbations on each component, the perturbation that yields the best improvement to the message length [as per Equation (4)] is chosen going into the next iteration, and so on, until convergence.

The rationale of each Split, Merge, and Delete operations together with the full details of their mechanics are provided in Supplementary Section S2. Furthermore, Supplementary Section S11 demonstrates the stability and convergence of this search process.

## 3 Results and discussion

### 3.1 Datasets and benchmarks

#### 3.1.1 Curating the dihedral angle datasets

Atomic coordinates of 38,895 protein structures with non-redundant amino acid sequences (≤50% sequence identity) were derived from the Protein Data Bank (Berman et al. 2000), considering only structures with an *R*-factor cut-off at 0.3 and resolution cut-off at 3.5 Å or better. We call this collection PDB50. Further, as a way to test the effect that precision of input data has on the inferred models, we also consider another (≤50% sequence identity) dataset containing 9568 high-resolution (≤1.8 Å) X-ray structures with a *B*-factor cut-off of 40 and *R*-factor cut-off of 0.22. We call this collection PDB50HighRes.

For a complete atomic coordinate record of each amino acid observed in any considered structure, we calculate a vector of backbone and sidechain dihedral angles: $\left\{\phi ,\psi ,\omega ,\chi_{1},\chi_{2},\ldots \right\}$. (We note that the partial double-bond characteristic of peptide bond makes *ω* typically $∼180^{{}^{\circ}}$ and rarely $∼0^{{}^{\circ}}$. Thus, for our inference, *ω* dihedrals were ignored from the input set.) Overall, this resulted in 22,177,093 observations (vectors of dihedral angles) from PDB50 and 3,774,207 observations for PDB50HighRes, considering only the atomic coordinates of 20 natural amino acids within proteins. We then partitioned these observations into 20 sets of amino acid specific dihedral angle vectors ($X^{\left(\text{aa}\right)}$), one for each distinct amino acid (aa).

Table 1 gives the breakdown of the number of observations per amino acid type, along with their corresponding number of (backbone + sidechain) dihedral angles. For each of these amino acid specific input sets $X^{\left(\text{aa}\right)}$, its corresponding mixture model $M(\Lambda^{\left(\text{aa}\right)})$ (one for PDB50 dataset and another for PDB50HighRes dataset) was inferred and their parameters estimated automatically using the MML methodology (described in Section 2).

**Table 1.** PDB50 dataset statistics: amino acid type (aa), number of observations of that amino acid in PDB50 ($N^{\left(\text{aa}\right)}$), and the total number of (backbone + sidechain) dihedral angles in that amino acid ($d^{\left(\text{aa}\right)}$).

aa	$N^{\left(\text{aa}\right)}$	$d^{\left(\text{aa}\right)}$	aa	$N^{\left(\text{aa}\right)}$	$d^{\left(\text{aa}\right)}$	aa	$N^{\left(\text{aa}\right)}$	$d^{\left(\text{aa}\right)}$
LEU	2,171,630	4	ASP	1,279,567	4	GLN	820,871	5
ALA	1,861,359	2	THR	1,221,604	3	TYR	788,176	4
VAL	1,601,058	3	LYS	1,176,395	6	HIS	515,611	4
GLY	1,588,115	2	ARG	1,130,448	7	MET	417,170	5
GLU	1,446,860	5	PRO	1,004,859	4	TRP	310,470	4
SER	1,337,273	3	ASN	948,274	4	CYS	296,547	3
ILE	1,333,508	4	PHE	927,298	4			

The counts in $d^{\left(\text{aa}\right)}$ ignore the *ω* dihedral angle.

#### 3.1.2 Dunbrack backbone-dependent rotamer libraries

We benchmark the performance and fidelity of our inferred mixture models against the latest version of the Dunbrack ‘backbone-dependent’ rotamer (sidechain conformation) libraries (Shapovalov and Dunbrack 2011), across varying degrees of smoothing [2%, 5% (default), 10% and 20%] that those libraries provide. The Dunbrack libraries define the state of the art for modelling and sampling sidechain conformations, ‘conditioned’ on any stated backbone dihedral angles 〈ϕ,ψ〉. Specifically, the Dunbrack rotamer library discretizes each amino acid’s backbone dihedral angles 〈ϕ,ψ〉 into $36^{2}=1296$ bins (of $10^{{}^{\circ}}\times 10^{{}^{\circ}}$ granularity). For each 〈ϕ,ψ〉 bin, there are commonly $3^{m}$ models. Here, 3 arises from the three-way discretization of each sidechain dihedral angle into {gauche+ (g+), trans (t), gauche- (g-)} states, whereas *m* denotes the number of ‘sidechain’ dihedral angles $\langle \chi_{1},\chi_{2},\ldots \rangle$ in that amino acid. For example, amino acid, methionine has *m *=* *3 and the Dunbrack rotamer library lists $36\times 36\times 3^{3}=34,992$ models across its 1296 possible 〈ϕ,ψ〉 bins. The Dunbrack rotamer library divides the set of amino acid types into ‘rotameric’ and ‘non-rotameric’ categories. The use of the closed-form computation of $3^{m}$ models holds for all ‘rotameric’ amino acids, whereas the ‘non-rotameric’ amino acids (glutamic acid, glutamine, aspartic acid, asparagine, tryptophan, histadine, tyrosine, and phenylalanine) have more components, as some of their sidechain dihedrals do not conform to three-way discretizations.

### 3.2 Information-theoretic complexity versus fidelity/fit of the inferred models

In almost all model selection problems, one seeks answers to two key questions: (i) What is the fidelity of the model in its ability to explain observed data? (ii) How complex is the selected model?. The second question is necessary for when there is a simpler model (in complexity terms) that can explain/fit the same data equivalently or better than a more complex model, then the simpler model is preferred not only due to Ockham’s razor, but also made rigorous by the Bayes theorem (Allison 2018).

The information-theoretic framework of MML provides a direct way to quantify model complexity and fit in terms of bits. For any proposed model, the total two-part message length combines (i) the lossless encoding of the model, the length (bits) of which yields the model’s (descriptive) complexity, and (ii) the lossless encoding of the observed data given that model, the length (bits) of which yields its fidelity by quantifying how well the model fits the data (see Section 2.2).

Table 2 gives the complexity and fidelity statistics of our inferred models and compares it directly with the state-of-the-art Dunbrack rotamer library at 5% (‘default’) smoothing level (see Supplementary Section S12 for results on other smoothing levels). Before we discuss these quantitative results, let us explore how/why they can be evaluated fairly, and on an equal footing.

**Table 2.** Quantitative comparison between the MML-inferred mixture model ($M^{\left(\text{aa}\right)}$) and that of the Dunbrack rotamer library ($D_{\;\text{rotamer}}^{\left(\text{aa}\right)}$).

		**MML mixture model (** $M^{\left(\text{aa}\right)}$ **) message length statistics in bits (rounded)**					**Dunbrack rotamer library (** $D_{\text{rotamer}}^{\left(\text{aa}\right)}$ **) message length statistics in bits (rounded)**					Null model (raw) in bits	
(aa)	$N^{\left(\text{aa}\right)}$	($\left|M^{\left(\text{aa}\right)}\right|$; $|\Lambda^{\left(\text{aa}\right)}|$)	First part (complexity)	Second part (fit)	Total (complexity + fit)	$\frac{\text{Total}}{N^{\left(\text{aa}\right)}}$	($\left|D_{\;\text{rotamer}}^{\left(\text{aa}\right)}\right|$; #Params)	First part (complexity)	Second part (fit)	Total (complexity + fit)	$\frac{\text{Total}}{N^{\left(\text{aa}\right)}}$	Null($X^{\left(\text{aa}\right)}$)	$\frac{\text{Null}(X^{\left(\text{aa}\right)})}{N^{\left(\text{aa}\right)}}$
LEU	2,171,630	(165; 1484)	7017	34,540,650	34,547,667	15.9	(11,664; 57,024)	1,079,722	46,109,408	47,189,130	21.7	53,595,177	24.7
ALA	1,861,359	(25; 124)	701	14,847,660	14,848,361	8.0	(N/A; N/A)	N/A	N/A	N/A	N/A	22,968,891	12.3
VAL	1,601,058	(96; 671)	3389	18,795,871	18,799,260	11.7	(3888; 10,368)	217,209	26,750,651	26,967,860	16.8	29,635,223	18.5
GLY	1,588,115	(30; 149)	746	15,965,309	15,966,055	10.1	(N/A; N/A)	N/A	N/A	N/A	N/A	19,597,101	12.3
GLU	1,446,860	(262; 2881)	12,205	33,234,644	33,246,849	23.0	(69,984; 488,592)	9,696,033	39,933,578	49,629,612	34.3	44,635,088	30.8
SER	1,337,273	(114; 797)	3825	18,289,465	18,293,291	13.7	(3888; 10,368)	210,730	23,624,303	23,835,033	17.8	24,752,622	18.5
ILE	1,333,508	(172; 1547)	7356	20,475,170	20,482,526	15.4	(11,664; 57,024)	964,619	27,688,670	28,653,289	21.5	32,910,577	24.7
ASP	1,279,567	(170; 1529)	6524	23,223,302	23,229,826	18.2	(23,328; 115,344)	2,336,817	27,634,793	29,971,610	23.4	31,579,330	24.7
THR	1,221,604	(90; 629)	3057	15,740,512	15,743,569	12.9	(3888; 10,368)	211,687	20,733,566	20,945,253	17.1	22,611,615	18.5
LYS	1,176,395	(266; 3457)	13,691	32,006,948	32,020,639	27.2	(104,976; 943,488)	14,337,386	37,818,245	52,155,632	44.3	43,549,614	37.0
ARG	1,130,448	(250; 3749)	15,898	32,987,603	33,003,501	29.2	(104,976; 943,488)	15,442,702	37,252,663	52,695,365	46.6	48,823,456	43.2
PRO	1,004,859	(231; 2078)	13,779	11,810,146	11,823,926	11.8	(2592; 11,664)	254,495	18,318,268	18,572,763	18.5	24,799,619	24.7
ASN	948,274	(180; 1619)	6793	17,855,829	17,862,622	18.8	(46,656; 231,984)	4,586,850	21,232,141	25,818,991	27.2	23,403,118	24.7
PHE	927,298	(226; 2033)	9365	15,950,596	15,959,961	17.2	(23,328; 115,344)	2,216,337	19,089,817	21,306,154	23.0	22,885,436	24.7
GLN	820,871	(239; 2628)	10,868	18,921,120	18,931,988	23.1	(139,968; 978,480)	18,417,683	23,167,291	41,584,974	50.7	25,323,563	30.8
TYR	788,176	(192; 1727)	7830	13,596,728	13,604,557	17.3	(23,328; 115,344)	2,248,951	16,184,209	18,433,160	23.4	19,451,947	24.7
HIS	515,611	(163; 1466)	6227	9,602,801	9,609,028	18.6	(46,656; 231,984)	4,373,651	11,419,682	15,793,334	30.6	12,725,125	24.7
MET	417,170	(270; 2969)	12,440	9,306,924	9,319,365	22.3	(34,992; 243,648)	4,222,664	11,504,102	15,726,767	37.7	12,869,538	30.8
TRP	310,470	(212; 1907)	8591	5,397,385	5,405,976	17.4	(46,656; 231,984)	4,062,897	6,659,922	10,722,819	34.5	7,662,306	24.7
CYS	296,547	(96; 671)	3148	3,943,308	3,946,457	13.3	(3,888; 10,368)	190,183	5,025,548	5,215,731	17.6	5,489,018	18.5

For each of the 20 naturally occurring amino acids (aa), $N^{\left(\text{aa}\right)}$ gives the size of the input set ($X^{\left(\text{aa}\right)}$) on which the comparison is based. $\left|M^{\left(\text{aa}\right)}\right|$ gives the number of components of the mixture model, and $\left|\Lambda^{\left(\text{aa}\right)}\right|$ gives the number of parameters across all components of the mixture model, inferred unsupervised. $\left|D_{\;\text{rotamer}}^{\left(\text{aa}\right)}\right|$ is the cumulative sum of all components described by the Dunbrack rotamer library, whereas #Params gives the corresponding total number of parameters implicit in their library. Across both models, the complexity (first part length in bits), fidelity (second part length in bits), and their two-part total are shown. The number of bits-per-residue for each of the models is also shown (the respective total message length by $N^{\left(\text{aa}\right)}$). Finally, to measure the extent of lossless compression each model provides, the null model message length of stating the vector of dihedral angles encoded under a uniform distribution is shown as a bottom-line. Note the ‘N/A’ terms across alanine (ALA) and glycine (GLY) arise because those amino acids do not have sidechain dihedral angles. While we model the joint distributions of dihedral including the backbone, Dunbrack on the other hand only provide sidechain distributions conditional on the backbone. Hence for ALA and GLY, Dunbrack library estimates are necessarily empty.

For each of the 1296 bins in the Dunbrack library, the information in their library can be directly translated as a bin-wise mixture model with a fixed number of mixture components, where each component contains a product of *m* von Mises circular distributions, and *m* is the number of sidechain dihedral angles for the specified amino acid (aa). [We note that amino acids alanine (ALA) and glycine (GLY) have no sidechain dihedral angles, so the Dunbrack library do not have any models for ALA and GLY.] However, as mentioned above, the number of components of the each of those 1296 mixture models related to an amino acid is static/fixed and corresponds to the number of discrete states over *m* sidechain angles (often three-way for each sidechain dihedral angle *χ*, as discussed earlier). Thus, the number of mixture components for each of the 〈ϕ,ψ〉 bin is usually $3^{m}$ which yield a large number of models across all bins (e.g. 34,992 for methionine as shown in Table 2). This number matters, as it is proportional to the number of von Mises parameters (and respective mixtures’ weights) that informs the complexity of the statistical model being proposed. In contrast, the MML mixture model infers only one mixture model for any amino acid, jointly over all (backbone + sidechain) dihedral angles with all of its mixture parameters estimated unsupervised, including the number of mixture components $\left|M^{\left(\text{aa}\right)}\right|$.

Comparing the model fit/fidelity is more involved: while our work models the joint distributions over all (backbone + sidechain) dihedral angles, Dunbrack’s only deals with sidechain dihedrals conditioned on discretized states of the backbone. With this difference in the models, there are two possible directions to take to ensure the comparison of fidelity between the two is on the same footing. For any set of observations of all dihedral angles for a specified amino acid $X^{\left(\text{aa}\right)}$:

1. The ϕ and *ψ* under Dunbrack model are stated over a uniform distribution—for this is precisely their underlying model—so that the message length of stating each vector of dihedrals using both models can be objectively compared. We show these results for PDB50 in the main text (see Table 2). Results for PDB50HighRes are included in Supplementary Section S4.
2. From each MML-inferred mixture model, we drop/omit the von Mises circular terms corresponding to backbone dihedral angles when estimating the length, yielding the second part of the message for only the sidechain dihedral angles of the observations. These results are presented in Supplementary Sections S3 (for PDB50) and S5 (for PDB50HighRes).

The above two ways of comparing the fidelity of the two models yield a similar conclusion: the MML-inferred mixture models (across all amino acid) are not only significantly more concise, but also explain the observed data better than the Dunbrack rotamer library (across the levels of smoothing they provide). Supplementary Section S9 provides a detailed explanation of how the lossless message length terms for Dunbrack’s model are calculated.

Comparing the model complexity, Table 2 clearly shows that MML-inferred models are three orders of magnitude (in bits) more concise than those of the Dunbrack rotamer library. This is mainly due to the proliferation of the number of parameters in the Dunbrack model (see the eighth column of Table 2 under #nParams) compared with the lower number in the MML mixture model (third column under $\left|\Lambda^{\left(\text{aa}\right)}\right|$).

Further, comparing the model fidelity, all MML mixture models yield a better (lossless) explanation of the observed data than the corresponding Dunbrack models. The improvement varies with amino acids with most improvement observed for proline (PRO) where the second-part message length from MML mixture model is ∼35% shorter than Dunbrack. On the other end, for arginine (ARG) the improvement is ∼11%. The median improvement is  ∼18% for glutamine (GLN). The mean sits at 20.1% improvement on PDB50 and 19.3% on PDBHighRes (Supplementary Table S2). Thus, from the results, it can be unambiguously concluded that the MML mixture models from this work outperform the state of the art in an objective quantitative comparison. Supplementary Sections S3 and S5 provide the alternative comparison between complexity and fit of the two models, involving the lossless comparison of sidechain dihedral angles and ignoring the backbone for PDB50 and PDB50HighRes.

Finally, we also assess how similar/different the inferred MML mixture models are across individual amino acids on the two datasets we have considered: PDB50 and PDB50HighRes. We use the measure of Kullback–Leibler (KL) relative entropy divergence that provides a direct way to compare two probability distributions. Supplementary Table S4 provides the KL-divergence values. The small KL-divergence across all amino acids indicates the proximity/similarity of the two inferred distributions. More generally, it has been demonstrated that the MML estimator is statistically robust to detect signal reliably even when the precision of input data varies (Wallace 2005).

### 3.3 Visualization of fidelity of the models

Here, we compare the fidelity of MML mixture models and Dunbrack rotamer library by randomly sampling 100,000 data points (vectors of dihedral angles) and contrasting the resultant distributions from the two models against the observed (empirical) distribution. The method of sampling from any MML-inferred mixture model and (for comparison) Dunbrack’s library is described in Supplementary Section S10.

To be able to assess similarities and differences visually, we examine two specific amino acids, methionine (MET) and glutamine (GLN). We choose these pairs because (i) they both have three sidechain angles $\langle \chi_{1},\chi_{2},\chi_{3}\rangle$, thus allowing their joint visualizations in 3D and (ii) MET falls into the ‘rotameric’ class of amino acids, whereas GLN falls into the ‘non-rotameric’ class (Shapovalov and Dunbrack 2011), hence providing a representation from those two classes for inspection.

Below we show these qualitative comparisons for the models inferred on the PDB50 dataset. The corresponding ones for PDB50HighRes are included in Supplementary Section S6.

**Figure 2.**
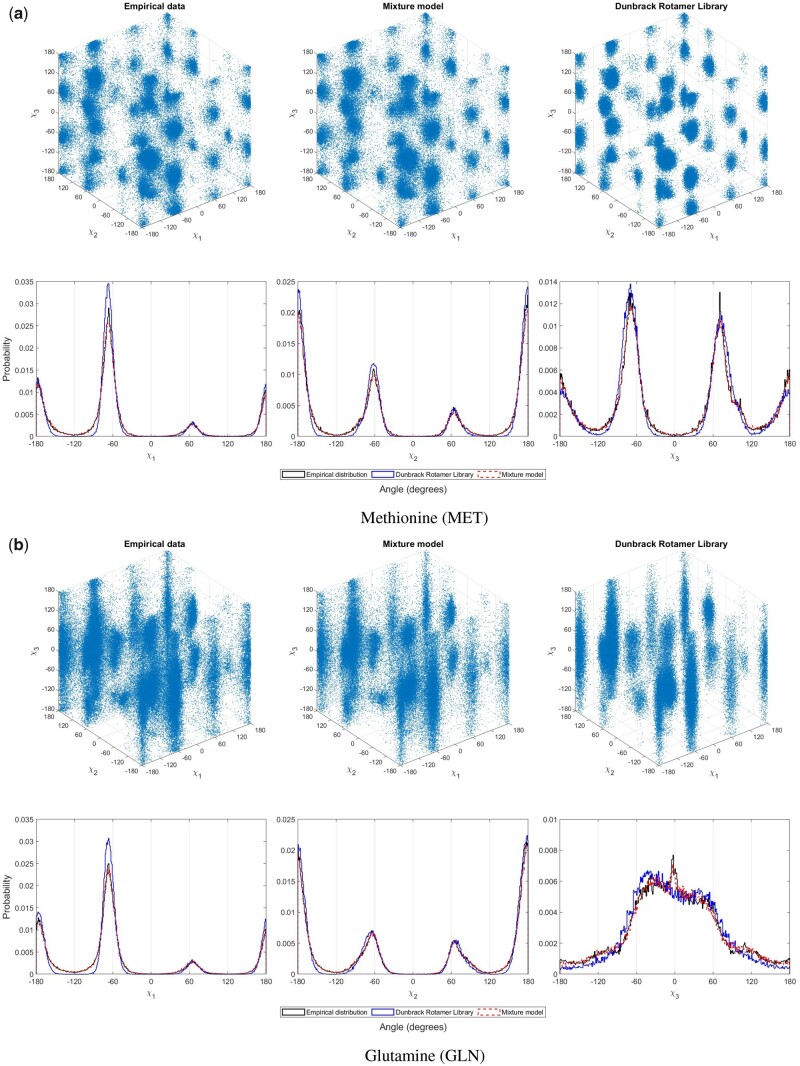
(a) The projection, into the sidechain $\left(\chi_{1},\chi_{2},\chi_{3}\right)$ space (unwrapped), of 100,000 randomly sampled points (vector of dihedral angles) for the amino acid methionine (MET) from MML mixture model (first row, center), of the same number of points from the Dunbrack model (first row, right), and of the observed (empirical) distribution of the same angles (first row, left). In the plots of the second row, the same data are visualized differently over three separate plots, with each of the three sidechain dihedral angles as *x*-axis (unwrapped), with *y*-axis showing the corresponding relative probabilities (in a $1^{{}^{\circ}}$ intervals). (b) The third and fourth rows plots are similar to first and second, respectively, but for the ‘non-rotameric’ amino acid, glutamine (GLN).

Figure 2 clearly shows that the sampled points/vectors from the MML-inferred mixture model for both these cases are significantly closer to the empirical distribution of those respective amino acids than the points/vectors randomly sampled from the Dunbrack library, which are comparatively sparser. Although the sampled points cover the main rotameric preferences, they do fall short in modelling the details of the spread seen in the empirical distribution, which the MML mixture model does well in explaining. This visualization is a qualitative demonstration of the clear quantitative difference we observed in their second part message length terms (which quantifies fidelity/fit in bits of information) shown earlier in Table 2: MET (19.1% difference) and GLN (18.3%). We already saw that the complexity (first) part of these models are orders of magnitude different (in bits), again in favour of the MML mixture model. This in itself demonstrates the power of inference made under the MML framework, and the natural trade-off between complexity and fit the framework permits. It is also a demonstration of the effectiveness of the EM method employed to infer these mixtures.

**Figure 3.**
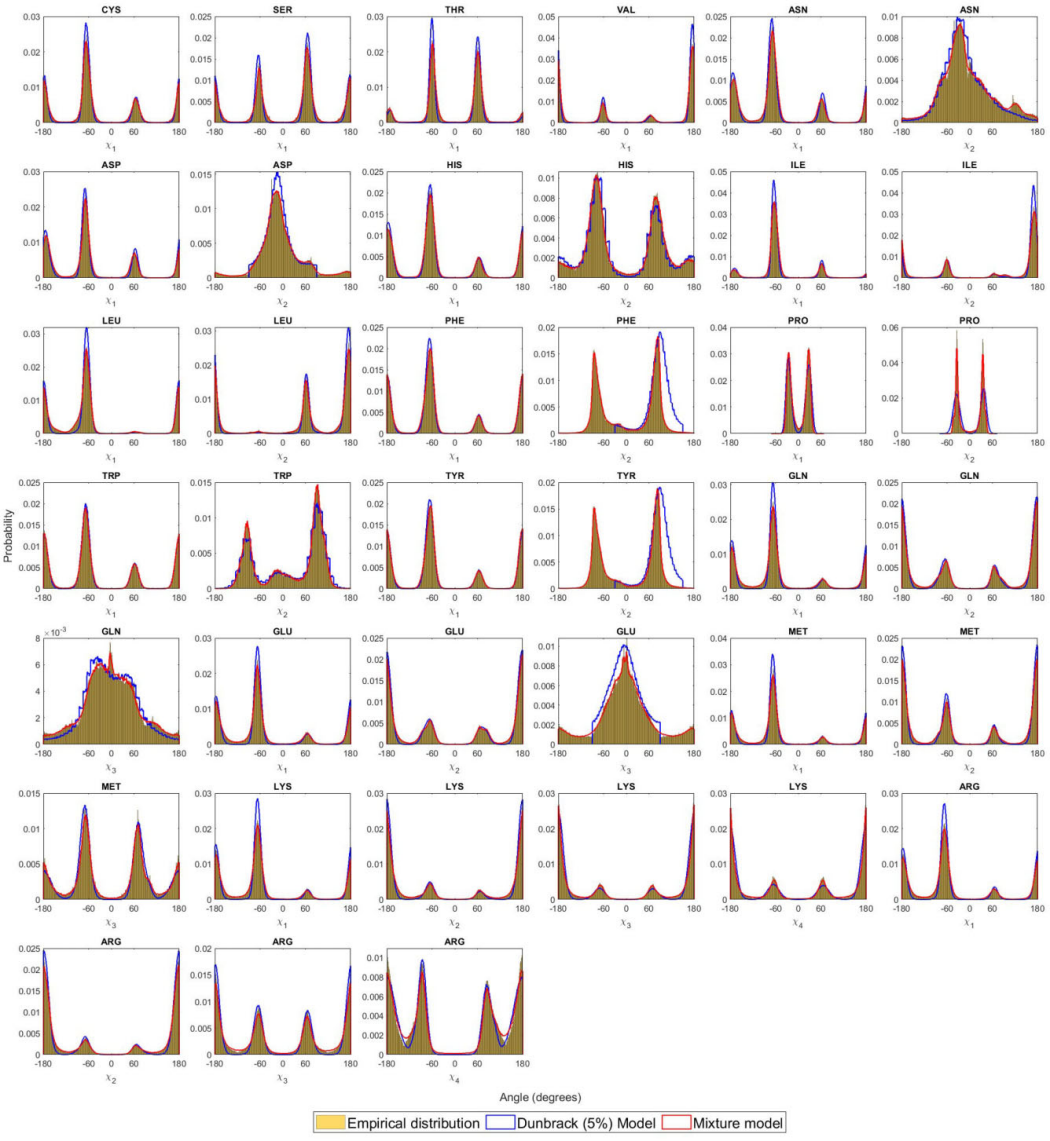
Fidelity of the inferred MML mixture models: the projected distribution of individual sidechain dihedral angles across all amino acids derived by randomly sampling $N^{\left(\text{aa}\right)}$ datapoints (see Table 1) from MML derived mixture models and Dunbrack (5% smoothed) library, and compared with the empirical distribution.

Finally, to give an overall view of the qualitative differences across all amino acids, we plot the probability distribution for each sidechain angle for which the MML mixture model can project onto the respective dihedral angle dimension, and compare it against the empirical (observed) distribution of that angle. For each amino acid, we randomly sample data points (vector of dihedral angles) from mixture models and plot against the corresponding empirical distribution. Figure 3 shows these plots across all amino acids, with the mixture model shown as a red curve, and the empirical distribution shown in yellow. For comparison, we include the distribution of sidechain dihedral angles by randomly sampling from the Dunbrack library across amino acids, shown in the same figure (in blue). The plots show that our mixture models fit better the empirical distribution than the Dunbrack models. (The visualization for PDB50HighRes is provided in Supplementary Section S7, and follows the same conclusions as above.)

## 4 Conclusion

We have successfully modelled the joint distribution of mainchain and sidechain dihedral angles of amino acids using mixture models. By measuring the Shannon information content, we showed that our mixture models outperform the models implied by the Dunbrack rotamer libraries (across levels of smoothing), both in terms of its model complexity (by three orders of magnitude) and its fidelity (yielding on average 20% more lossless compression) when explaining the observed dihedral angle datasets with varying resolution and filtering thresholds. We also demonstrated the robustness of the MML method of estimation, and show that the inferred mixture models are not prone to the pitfalls of under/over-fitting and other inconsistencies common to many statistical model selection exercises. The brevity of our mixture models also provide computationally cheap and reliable way to sample jointly $\langle \phi ,\psi ,\chi_{1},\chi_{2},\ldots \rangle$ dihedral angles (and also conditionally given 〈ϕ,ψ〉) and are ready for use in downstream studies: experimental structure refinement, *de novo* protein design, protein structure prediction, among others. Our mixture models, PhiSiCal (ϕψχal), are available for download from http://lcb.infotech.monash.edu.au/phisical. Also available from this link are programs to sample from the mixture models and report descriptive statistics (probability, log-odds ratios between pairs of models, null probability to estimate statistical significance, etc.) for use in modelling and simulation exercises.

We foresee several applications of candidate samples of amino acid conformations generated from PhiSical models. These include computational support to model amino acid 3D coordinates into electron density maps, predicting sidechain conformations given backbone states of amino acids, assessing protein structures to detect conformation-outliers, driving perturbations in molecular dynamic simulations, among others. We aim to address these as future work.

## Supplementary Material

btad251_Supplementary_Data
