# Supplementary material for: Getting ‘ϕψχal’ with proteins: minimum message length inference of joint distributions of backbone and sidechain dihedral angles
Source: Bioinformatics. 2023 Jun 30;39(Suppl 1):i357–67. doi: 10.1093/bioinformatics/btad251 (PMC10311319; doi:10.1093/bioinformatics/btad251)
Supplement: btad251_Supplementary_Data [file btad251_supplementary_data.pdf]

Supplementary Material for

Getting '$\phi\psi\chi$al' with proteins: minimum message length inference of joint distributions of backbone and sidechain dihedral angles

Piyumi R. Amarasinghe[1], Lloyd Allison[1], Peter J. Stuckey[1,2], Maria Garcia de la Banda[1,2], Arthur M. Lesk[3], and Arun S. Konagurthu[1,*]

[1]Department of Data Science and Artificial Intelligence, Faculty of Information Technology, Monash University, Clayton, VIC 3800, Australia
[2]OPTIMA ARC Industrial Training and Transformation Centre, Carlton, VIC 3053, Australia
[3]Department of Biochemistry and Molecular Biology, Pennsylvania State University, University Park, PA 16802, USA

Supplementary data is available to download from:
`PhiSiCal` ($\phi\psi\chi$al), http://lcb.infotech.monash.edu.au/phisical

S1 MML estimation for concentration parameter

We have seen in the main text (section 2.3.2, equation 9) how the mean (μ) parameter is estimated for each von Mises component in the mixture. Here, we will provide details of the numerical estimation of the concentration parameter (κ). By using the Wallace-Freeman method (Wallace and Freeman, 1987), the total message length for stating N observations of circular random variables x, where $x \in (-\pi, \pi]$ using a von Mises distribution can be derived as follows. Let $x \in X$ and $X = \{x_1, x_2, ..., x_N\}$ are the N observations of x. Further consider the von Mises distribution $f(x; \langle\mu, \kappa\rangle)$ with mean $\mu \in (-\pi, \pi)$ and concentration $\kappa > 0$. The associated net message $I(\langle\mu, \kappa\rangle, X)$ can be approximated as,

$$I(\langle\mu,\kappa\rangle, X) \approx \log(q_2) + \log\left(\frac{\sqrt{\det(\mathcal{F}(\langle\mu,\kappa\rangle))}}{h(\langle\mu,\kappa\rangle)}\right) + \mathcal{L}(\langle\mu,\kappa\rangle) - 2N\log(\epsilon) + 1 \quad (1)$$

Therefore, optimal concentration parameter κ_{MML} that minimizes Equation 1, can be derived by

$$\kappa_{MML} = \underset{\kappa}{\operatorname{argmin}}\, I(\langle\mu,\kappa\rangle, X) \quad (2)$$

As $\frac{\partial I(\langle\mu,\kappa\rangle, X)}{\partial\kappa} = 0$ results in a non-linear equation without a closed-form solution, we use the Newton–Raphson method as a reasonable approximation for finding roots of Equation 2 (Kasarapu and Allison, 2015).

Let $G(\kappa) = \frac{\partial I(\langle\mu,\kappa\rangle, X)}{\partial\kappa}$, then κ_{MML} can be approximated by twice iteration of Newton-Rapson method and using initial guess of roots $\kappa_0 = \kappa_B$. Here κ_B is the *Banerjee's approximation* (Banerjee *et al.*, 2005) for the concentration parameter which can be used as a feasible starting point for approximating roots of $G(\kappa) = 0$. Let κ_1, κ_{MML} correspond to the roots approximated at the first two iterations of the Newton-Rapson method,

$$\kappa_1 = \kappa_B - \frac{G(\kappa_B)}{G'(\kappa_B)} \quad \text{and} \quad \kappa_{MML} = \kappa_1 - \frac{G(\kappa)}{G'(\kappa)} \quad (3)$$

Here κ_B is evaluated by,

$$\kappa_B = \frac{\bar{R}(2 - \bar{R}^2)}{(1 - \bar{R}^2)} \text{ where } \bar{R} = \frac{||R||}{N} \quad (4)$$

R is the vector sum of each x circular variable and $||R||$ is the vector norm of the resultant vector R. We use κ_{MML} as the approximation for concentration parameter minimizing $I(\langle\mu,\kappa\rangle, X)$.

S2 Searching for optimal mixture

This algorithm employs the MML paradigm to quantify the fitness of competing statistical models (see main text for a detailed explanation of the MML model selection paradigm). In a nutshell, under MML, an optimal model is the one that yields the minimum two-part message length over all possible competing models. This remains a hard optimization problem. We employ a Expectation-Maximization (EM) based approach, which is commonly used for unsupervised statistical parameter estimation problems.

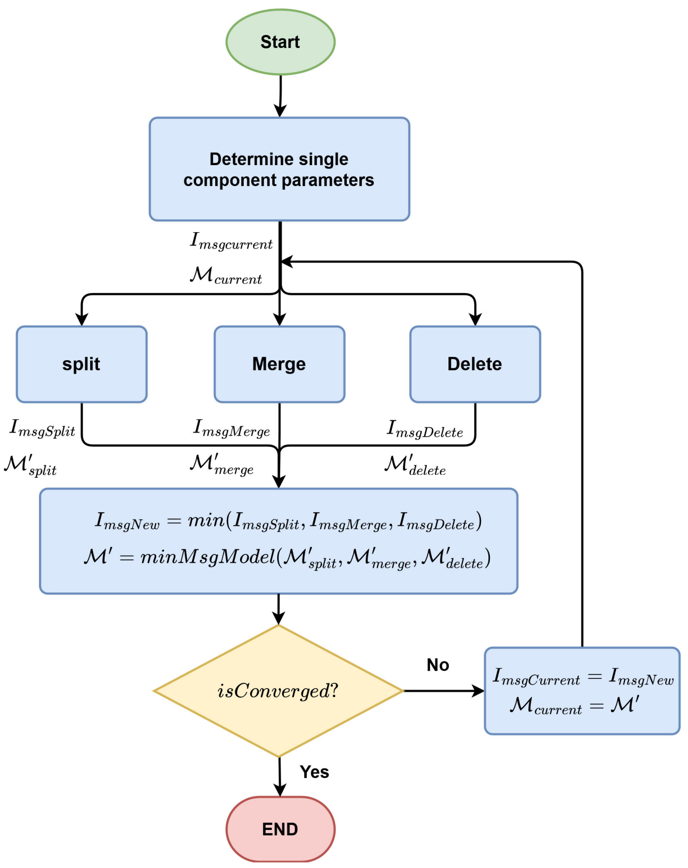

Figure SF 1. Flow chart representing the functional flow of the search algorithm

The conceptual flow of the EM search is shown in Fig SF 1. The EM starts with a single component mixture model ($|\mathcal{M}| = 1$) at iteration $t = 0$, whose parameters are estimated as described in section S1. Starting from this single component mixture, the model undergoes a series of *Split*, *Merge* and *Delete* perturbations, chosen deterministically and greedily to improve the total message length objective. Each operation and their rationale is described below. For this, assume that at an arbitrary iteration t, we have a K component mixture model ($|\mathcal{M}| = K$).

S2.1 Split operation:

Given a K component mixture, the primary goal of this operation is to find two distinct sub-populations in the current mixture such that the new mixture with $K+1$ components is capable of better explaining the data. Consider an arbitrary $\mathcal{M}_j$ component ready for split operation. This $\mathcal{M}_j$ component (*parent*) is split into two new components (*children*). The resulting sub-mixture is then optimized using the EM algorithm while the other $(K-1)$ components are untouched. Let $\mathcal{M}_j^a, \mathcal{M}_j^b$ be child components of $\mathcal{M}_j$.

S2.1.1 Initialize child components.

EM algorithm is sensitive to initial parameter values. As the message length (or likelihood) function of a mixture model is not unimodal, depending on the starting parameters of the mixture, it could get trapped in a sub-optimal solution. One could execute the EM algorithm starting from a variety of initial parameter values chosen randomly to avoid a poor approximation to the true minimum (for message length as the objective function). However, this does not ensure the accuracy of the resulting estimates. In order to select the initial parameters of the sub-mixture, we consider the distribution of membership among the probable child components.

Given $\Theta_j = \{\langle \mu_{j_p}, \kappa_{j_p} \rangle\}_{\forall 1 \le p \le d}$ of the parent component, we can locate two starting mean values for each dihedral-angle p by selecting two points (in $(-\pi, \pi]$ space) which are one standard deviation away on either side of Θ_j. These new mean values in the vicinity of the parent's mean can serve as starting parameters of the child components resulting from *splitting* the parent component. For a d-dimensional datum, there are (2^d) potential split combinations. But the split could be required in only a few directions while other mean directions need unchanged. Hence altogether there are $(3^d - 1)$ split combinations that can be assigned as initial mean values. Executing the EM algorithm on all the combinations to select the optimal sub-mixture is computationally expensive. Due to that, we calculate the membership (refer to Equation (7) in the main text) under each plausible split combination and select the two combinations producing the highest membership. The corresponding mean values serve as good starting values of the mean direction of the two-component sub-mixture. Furthermore, the concentration parameters of the parent component are used as starting concentration values and weights are equally distributed among children.

Once child components are initialized, the EM algorithm is executed on the two-component sub-mixture.

S2.1.2 E-step

Let r_{ij}^a, r_{ij}^b be responsibilities of each child on datum $x_i \in X$ and n_j^a, n_j^b be memberships of $\mathcal{M}_j^a, \mathcal{M}_j^b$ child components. These values are calculated from Equation (7) in the main text.

S2.1.3 M-step

Given the responsibilities and memberships calculated at E-step, new parameters of the child components are calculated as follows. Let r_{ij} be the responsibility of parent component $\mathcal{M}_j$ on x_i.

$$w_j^a(t+1) = \frac{n_j^a + \frac{1}{2}}{N + \frac{1}{2}} \tag{5}$$

$$\mu_{j_p}^a = \frac{R_{j_p}^a}{||R_{j_p}^a||} \tag{6}$$

where $R_{j_p}^a$ is the vector sum of each $x_{i_p} \in X$ weighted by corresponding responsibilities r_{ij} and r_{ij}^a, similarly $||R_{j_p}^a||$ is the vector norm of the resultant vector $R_{j_p}^a$. Finally, concentration parameter $\kappa_{j_p}^a$,($\forall 1 \le p \le d$) is calculated by,

$$\bar{R}_{j_p}^a = \frac{||R_{j_p}^a||}{\sum_{i=1}^{N} r_{ij} r_{ij}^a} \tag{7}$$

The E-step and M-step are executed repeatedly until there is no *sufficient* gain in the message lengths between consecutive iterations. Once the sub-mixture is optimized, it is integrated into the $(K-1)$ mixture such that the $\mathcal{M}_j$ component is replaced by its successors. The resulting $(K+1)$ mixture is then optimized again by the EM algorithm to tune the parameters of the new mixture. The split operation is performed on every K element in the mixture, and only the $(K+1)$ component combination having the minimum two-part message length is selected to proceed (see Figure SF 1). Let $\mathcal{M}'_{split}$ be the selected new mixture at split operation.

S2.2 Merge operation:

Primary goal of Merge perturbation is to join components in a $K(K > 1)$ component mixture and explore how the resulting $(K-1)$ mixture performs. We consider Kullback-Leibler Distance (Kullback and Leibler, 1951) as a potential heuristic to identify components that are plausible to join. A component M_j is merged with another component with the minimum KL distance among the remaining $(K-1)$ components. During the merging, the responsibilities of one component are handed over to the other component resulting in a $(K-1)$ component mixture. The resulting $(K-1)$ component mixture is then tuned from the EM algorithm to readjust the component parameters. The merge operation is performed on every K element in the mixture exhaustively such that, only the $(K-1)$ component combination having the minimum two-part message length is selected to proceed. Let $\mathcal{M}'_{merge}$ be the selected new mixture at merge operation.

S2.2.1 KL Distance of a von Mises Distribution

Let $Q(x; \langle \mu_q, \kappa_q \rangle)$, $R(x; \langle \mu_r, \kappa_r \rangle)$ be two von Mises distributions then,

$$\begin{aligned} D_{KL}(Q \parallel R) &= \int_x Q(x) \log\left(\frac{Q(x)}{R(x)}\right) dx \\ &= \log\left(\frac{B_0(\kappa_r)}{B_0(\kappa_q)}\right) + A(\kappa_q)(\kappa_q - \kappa_r \cos(\mu_q - \mu_r)) \end{aligned} \tag{8}$$

Similar to usage in the main text, the modified Bessel function of order 0, I_0 is stated as B_0 in Equation 8.

By using Equation 8, the KL distance between two joint von Mises distributions $U(x; \Theta_u), V(x; \Theta_v)$ where $\Theta_u = \{\langle \mu_{u_p}, \kappa_{u_p} \rangle\}$ and $\Theta_v = \{\langle \mu_{v_p}, \kappa_{v_p} \rangle\}$, $(\forall 1 \le p \le d)$ is

$$D_{KL}(U \parallel V) = \sum_{p=1}^{d} \log\left(\frac{B_0(\kappa_{v_p})}{B_0(\kappa_{u_p})}\right) + A(\kappa_{u_p})(\kappa_{u_p} - \kappa_{v_p} \cos(\mu_{u_p} - \mu_{v_p})) \tag{9}$$

S2.3 Delete operation:

Given the greedy nature of the merge (which, for every component, always explores merging with the closest-neighbouring component in the current mixture), it becomes necessary to include a delete operation to remove any component that although being redundant escapes the greedy merge. Thus the delete operation aims to remove components from a $K(K > 1)$ mixture, one at a time and redistribute the responsibilities of the deleted component to the remaining components. The resulting $(K-1)$ mixture is then optimized using the EM algorithm to check whether an improved model could be achieved. The delete operation is performed on every K element in the mixture exhaustively such that, only the $(K-1)$ component combination having the minimum two-part message length is selected to proceed. Let M'_{delete} be the selected new mixture at the delete operation.

On completion of all perturbations, now we have $\mathcal{M}'_{split}, \mathcal{M}'_{merge}, \mathcal{M}'_{delete}$ mixtures which are the best choices from each perturbation. Finally, the model with the minimum two-part message length is selected as the starting mixture ($\mathcal{M}'$) of the next iteration. This process is continued repeatedly until the gains are minimal.

Refer (Kasarapu and Allison, 2015) for a detailed explanation of the perturbations.

S3 Quantitative comparison of message lengths for stating amino acid sidechain dihedral angles from PDB50 dataset

Table ST 1. This table provides a quantitative comparison between the MML-inferred mixture model ($\mathcal{M}^{(\text{aa})}$) and that of Dunbrack rotamer library ($\mathcal{D}^{(\text{aa})}_{\text{rotamer}}$) when explaining the PDB50 dataset. We emphasize that the reported message length terms are for losslessly stating only the sidechain dihedral angles of individual amino acids (aa) and do not consider the backbone dihedral angles $\langle \phi, \psi \rangle$. The 'N/A' terms across Alanine (ALA) and Glycine (GLY) arise because those amino acids do not have sidechain dihedral angles.

		MML Mixture Model ($\mathcal{M}^{(\text{aa})}$) message length statistics in bits (rounded)					Dunbrack Rotamer Library ($\mathcal{D}^{(\text{aa})}_{\text{rotamer}}$) message length statistics in bits (rounded)					Null Model (Raw) in bits	
(aa)	$N^{(\text{aa})}$	($\lvert\mathcal{M}^{(\text{aa})}\rvert$, $\lvert\Lambda^{(\text{aa})}\rvert$)	first-part (complexity)	second-part (fit)	Total (complexity+fit)	$\frac{\text{Total}}{N^{(\text{aa})}}$	($\lvert\mathcal{D}^{(\text{aa})}_{\text{rotamer}}\rvert$; #Params)	first-part (complexity)	second-part (fit)	Total (complexity+fit)	$\frac{\text{Total}}{N^{(\text{aa})}}$	Null($X^{(\text{aa})}$)	$\frac{\text{Null}(X^{(\text{aa})})}{N^{(\text{aa})}}$
LEU	2,171,630	(165;1,484)	4,095	17,578,246	17,582,342	8.1	(11,664;57,024)	1,079,717	41,766,148	42,845,865	19.7	26,797,588	12.3
ALA	1,861,359	(25;124)	N/A	N/A	N/A	N/A	(N/A;N/A)	N/A	N/A	N/A	N/A	N/A	N/A
VAL	1,601,058	(96;671)	1,625	6,607,950	6,609,575	4.1	(3,888;10,368)	217,204	23,548,535	23,765,739	14.8	9,878,408	6.2
GLY	1,588,115	(30;149)	N/A	N/A	N/A	N/A	(N/A;N/A)	N/A	N/A	N/A	N/A	N/A	N/A
GLU	1,446,860	(262;2,881)	7,754	21,695,912	21,703,666	15.0	(69,984;488,592)	9,696,028	37,039,858	46,735,886	32.3	26,781,053	18.5
SER	1,337,273	(114;797)	1,757	6,592,674	6,594,431	4.9	(3,888;10,368)	210,725	20,949,757	21,160,482	15.8	8,250,874	6.2
ILE	1,333,508	(172;1,547)	4,346	10,688,100	10,692,445	8.0	(11,664;57,024)	964,613	25,021,654	25,986,267	19.5	16,455,289	12.3
ASP	1,279,567	(170;1,529)	3,640	12,462,677	12,466,317	9.7	(23,328;115,344)	2,336,812	25,075,659	27,412,471	21.4	15,789,665	12.3
THR	1,221,604	(90;629)	1,445	5,531,460	5,532,905	4.5	(3,888;10,368)	211,682	18,290,358	18,502,040	15.1	7,537,205	6.2
LYS	1,176,395	(266;3,457)	9,833	22,078,096	22,087,929	18.8	(104,976;943,488)	14,337,381	35,465,455	49,802,836	42.3	29,033,076	24.7
ARG	1,130,448	(250;3,749)	12,164	23,504,690	23,516,854	20.8	(104,976;943,488)	15,442,697	34,991,767	50,434,464	44.6	34,873,897	30.8
PRO	1,004,859	(231;2,078)	8,956	5,257,024	5,265,980	5.2	(2,592;11,664)	254,490	16,308,550	16,563,040	16.5	12,399,809	12.3
ASN	948,274	(180;1,619)	3,703	9,582,207	9,585,910	10.1	(46,656;231,984)	4,586,845	19,335,593	23,922,438	25.2	11,701,559	12.3
PHE	927,298	(226;2,033)	5,401	8,460,779	8,466,181	9.1	(23,328;115,344)	2,216,332	17,235,221	19,451,553	21.0	11,442,718	12.3
GLN	820,871	(239;2,628)	6,804	12,270,999	12,277,803	15.0	(139,968;978,480)	18,417,678	21,525,549	39,943,227	48.7	15,194,138	18.5
TYR	788,176	(192;1,727)	4,480	7,193,098	7,197,578	9.1	(23,328;115,344)	2,248,946	14,607,857	16,856,803	21.4	9,725,974	12.3
HIS	515,611	(163;1,466)	3,443	5,175,762	5,179,204	10.0	(46,656;231,984)	4,373,646	10,388,460	14,762,106	28.6	6,362,562	12.3
MET	417,170	(270;2,969)	7,919	5,954,095	5,962,013	14.3	(34,992;243,648)	4,222,659	10,669,762	14,892,422	35.7	7,721,723	18.5
TRP	310,470	(212;1,907)	4,816	2,958,040	2,962,856	9.5	(46,656;231,984)	4,062,892	6,038,982	10,101,874	32.5	3,831,153	12.3
CYS	296,547	(96;671)	1,433	1,389,186	1,390,619	4.7	(3,888;10,368)	190,178	4,432,454	4,622,632	15.6	1,829,673	6.2

S4 Quantitative comparison of message lengths for stating amino acid (backbone + sidechain) dihedral angles from PDB50HighRes dataset

Table ST 2. This table provides a quantitative comparison between the MML-inferred mixture model ($\mathcal{M}^{(\text{aa})}$) and that of Dunbrack rotamer library ($\mathcal{D}^{(\text{aa})}_{\text{rotamer}}$) for stating dihedral angles (backbone + sidechain) of each of the twenty naturally occurring amino acids (aa). The the 'N/A' terms across Alanine (ALA) and Glycine (GLY) arise because those amino acids do not have sidechain dihedral angles. While we model the joint distributions of dihedral including the backbone, Dunbrack on the other hand only provides sidechain distributions conditional on the backbone. Hence ALA and GLY Dunbrack libraries are necessarily empty.

		MML Mixture Model ($\mathcal{M}^{(\text{aa})}$) message length statistics in bits (rounded)					Dunbrack Rotamer Library ($\mathcal{D}^{(\text{aa})}_{\text{rotamer}}$) message length statistics in bits (rounded)					Null Model (Raw) in bits	
(aa)	$N^{(\text{aa})}$	($\lvert\mathcal{M}^{(\text{aa})}\rvert$, $\lvert\Lambda^{(\text{aa})}\rvert$)	first-part (complexity)	second-part (fit)	Total (complexity+fit)	$\frac{\text{Total}}{N^{(\text{aa})}}$	($\lvert\mathcal{D}^{(\text{aa})}_{\text{rotamer}}\rvert$; #Params)	first-part (complexity)	second-part (fit)	Total (complexity+fit)	$\frac{\text{Total}}{N^{(\text{aa})}}$	Null($X^{(\text{aa})}$)	$\frac{\text{Null}(X^{(\text{aa})})}{N^{(\text{aa})}}$
LEU	343,752	(165;1,484)	6,788	5,012,392	5,019,181	14.6	(11,664;57,024)	950,779	6,587,654	7,538,433	21.9	8,483,696	24.7
ALA	334,111	(25;124)	666	2,533,968	2,534,634	7.6	(N/A;N/A)	N/A	N/A	N/A	N/A	4,122,880	12.3
GLY	294,278	(30;149)	706	2,851,845	2,852,551	9.7	(N/A;N/A)	N/A	N/A	N/A	N/A	3,631,346	12.3
VAL	274,596	(96;671)	3,261	2,971,624	2,974,885	10.8	(3,888;10,368)	192,834	4,163,843	4,356,677	15.9	5,082,710	18.5
GLU	238,682	(262;2,881)	6,304	3,867,073	3,873,377	16.2	(69,984;488,592)	8,509,192	6,189,754	14,698,946	61.6	7,363,250	30.8
ASP	227,558	(170;1,529)	3,671	2,862,306	2,865,978	12.6	(23,328;115,344)	2,062,259	4,632,995	6,695,254	29.4	5,616,063	24.7
SER	222,721	(114;797)	7,121	3,037,346	3,044,467	13.7	(3,888;10,368)	185,948	3,657,879	3,843,828	17.3	4,122,516	18.5
ILE	215,684	(172;1,547)	2,937	2,534,288	2,537,226	11.8	(11,664;57,024)	848,230	4,018,755	4,866,985	22.6	5,323,016	24.7
THR	212,562	(90;629)	13,332	5,049,521	5,062,854	23.8	(3,888;10,368)	187,511	3,295,033	3,482,543	16.4	3,934,475	18.5
LYS	195,868	(266;3,457)	15,558	5,307,530	5,323,088	27.2	(104,976;943,488)	12,526,749	5,878,978	18,405,727	94.0	7,250,945	37.0
ARG	188,400	(250;3,749)	13,482	2,014,498	2,027,980	10.8	(104,976;943,488)	13,518,806	5,758,992	19,277,798	102.3	8,136,897	43.2
PRO	177,534	(231;2,078)	11,853	5,161,336	5,173,189	29.1	(2,592;11,664)	225,394	3,195,740	3,421,135	19.3	4,381,486	24.7
ASN	162,196	(180;1,619)	6,554	2,908,746	2,915,300	18.0	(46,656;231,984)	4,025,549	3,472,766	7,498,315	46.2	4,002,949	24.7
PHE	153,192	(226;2,033)	9,063	2,540,627	2,549,690	16.6	(23,328;115,344)	1,940,884	3,077,402	5,018,286	32.8	3,780,733	24.7
GLN	136,703	(239;2,628)	10,547	2,986,890	2,997,437	21.9	(139,968;978,480)	16,100,149	3,615,527	19,715,676	144.2	4,217,236	30.8
TYR	134,950	(192;1,727)	7,576	2,254,154	2,261,731	16.8	(23,328;115,344)	1,970,652	2,718,877	4,689,528	34.8	3,330,526	24.7
HIS	89,382	(163;1,466)	6,013	1,609,829	1,615,841	18.1	(46,656;231,984)	3,818,112	1,928,561	5,746,674	64.3	2,205,921	24.7
MET	68,907	(270;2,969)	12,078	1,445,826	1,457,903	21.2	(34,992;243,648)	3,657,582	1,752,132	5,409,714	78.5	2,125,755	30.8
TRP	56,696	(212;1,907)	8,322	961,834	970,157	17.1	(46,656;231,984)	3,547,218	1,197,638	4,744,855	83.7	1,399,240	24.7
CYS	46,435	(96;671)	3,013	584,959	587,972	12.7	(3,888;10,368)	164,549	747,043	911,592	19.6	859,501	18.5

S5 Quantitative comparison of message lengths for stating amino acid sidechain dihedral angles from PDB50HighRes dataset

Table ST 3. This table illustrates a quantitative comparison between the MML-inferred mixture model ($\mathcal{M}^{(\text{aa})}$) and that of Dunbrack rotamer library ($\mathcal{D}^{(\text{aa})}_{\text{rotamer}}$) to state only sidechain dihedral angles of each of the twenty naturally occurring amino acids (aa). Here we have only considered the cost of stating the sidechain dihedral angles of each of the twenty naturally occurring amino acids (aa) and omitted the backbone $\langle \phi, \psi \rangle$. The 'N/A' terms across Alanine (ALA) and Glycine (GLY) arise because those amino acids do not have sidechain dihedral angles.

		MML Mixture Model ($\mathcal{M}^{(\text{aa})}$) message length statistics in bits (rounded)					Dunbrack Rotamer Library ($\mathcal{D}^{(\text{aa})}_{\text{rotamer}}$) message length statistics in bits (rounded)					Null Model (Raw) in bits	
(aa)	$N^{(\text{aa})}$	($\lvert\mathcal{M}^{(\text{aa})}\rvert$, $\lvert\Lambda^{(\text{aa})}\rvert$)	first-part (complexity)	second-part (fit)	Total (complexity+fit)	$\frac{\text{Total}}{N^{(\text{aa})}}$	($\lvert\mathcal{D}^{(\text{aa})}_{\text{rotamer}}\rvert$; #Params)	first-part (complexity)	second-part (fit)	Total (complexity+fit)	$\frac{\text{Total}}{N^{(\text{aa})}}$	Null($X^{(\text{aa})}$)	$\frac{\text{Null}(X^{(\text{aa})})}{N^{(\text{aa})}}$
LEU	343,752	(165;1,484)	3,872	2,460,581	2,464,453	7.2	(11,664;57,024)	950,774	5,900,150	6,850,924	19.9	4,241,848	12.3
ALA	334,111	(25;124)	N/A	N/A	N/A	N/A	(N/A;N/A)	N/A	N/A	N/A	N/A	N/A	N/A
GLY	294,278	(30;149)	N/A	N/A	N/A	N/A	(N/A;N/A)	N/A	N/A	N/A	N/A	N/A	N/A
VAL	274,596	(96;671)	1,502	1,001,833	1,003,334	3.7	(3,888;10,368)	192,829	3,614,651	3,807,480	13.9	1,694,237	6.2
GLU	238,682	(262;2,881)	7,407	3,387,681	3,395,088	14.2	(69,984;488,592)	8,509,187	5,712,390	14,221,577	59.6	4,417,950	18.5
ASP	227,558	(170;1,529)	3,424	2,088,202	2,091,626	9.2	(23,328;115,344)	2,062,254	4,177,879	6,240,133	27.4	2,808,032	12.3
SER	222,721	(114;797)	1,608	1,012,207	1,013,816	4.6	(3,888;10,368)	185,943	3,212,437	3,398,380	15.3	1,374,172	6.2
ILE	215,684	(172;1,547)	4,116	1,556,994	1,561,110	7.2	(11,664;57,024)	848,225	3,587,387	4,435,612	20.6	2,661,508	12.3
THR	212,562	(90;629)	1,331	861,198	862,528	4.1	(3,888;10,368)	187,506	2,869,909	3,057,414	14.4	1,311,492	6.2
LYS	195,868	(266;3,457)	9,480	3,482,352	3,491,831	17.8	(104,976;943,488)	12,526,744	5,487,242	18,013,986	92.0	4,833,963	24.7
ARG	188,400	(250;3,749)	11,829	3,806,227	3,818,056	20.3	(104,976;943,488)	13,518,801	5,382,192	18,900,993	100.3	5,812,069	30.8
PRO	177,534	(231;2,078)	8,664	932,851	941,515	5.3	(2,592;11,664)	225,389	2,840,672	3,066,061	17.3	2,190,743	12.3
ASN	162,196	(180;1,619)	3,470	1,575,926	1,579,395	9.7	(46,656;231,984)	4,025,544	3,148,374	7,173,918	44.2	2,001,474	12.3
PHE	153,192	(226;2,033)	5,104	1,356,899	1,362,003	8.9	(23,328;115,344)	1,940,879	2,771,018	4,711,896	30.8	1,890,366	12.3
GLN	136,703	(239;2,628)	6,489	1,944,633	1,951,122	14.3	(139,968;978,480)	16,100,144	3,342,121	19,442,265	142.2	2,530,342	18.5
TYR	134,950	(192;1,727)	4,232	1,201,580	1,205,812	8.9	(23,328;115,344)	1,970,647	2,448,977	4,419,623	32.8	1,665,263	12.3
HIS	89,382	(163;1,466)	3,233	871,873	875,106	9.8	(46,656;231,984)	3,818,107	1,749,797	5,567,904	62.3	1,102,960	12.3
MET	68,907	(270;2,969)	7,561	916,757	924,319	13.4	(34,992;243,648)	3,657,577	1,614,318	5,271,895	76.5	1,275,453	18.5
TRP	56,696	(212;1,907)	4,552	531,702	536,255	9.5	(46,656;231,984)	3,547,212	1,084,246	4,631,458	81.7	699,620	12.3
CYS	46,435	(96;671)	1,303	203,080	204,383	4.4	(3,888;10,368)	164,544	654,173	818,717	17.6	286,500	6.2

S6 Qualitative comparison of model fit for methionine(MET) and glutamine(GLN) sidechain dihedral angles for PDB50HighRes

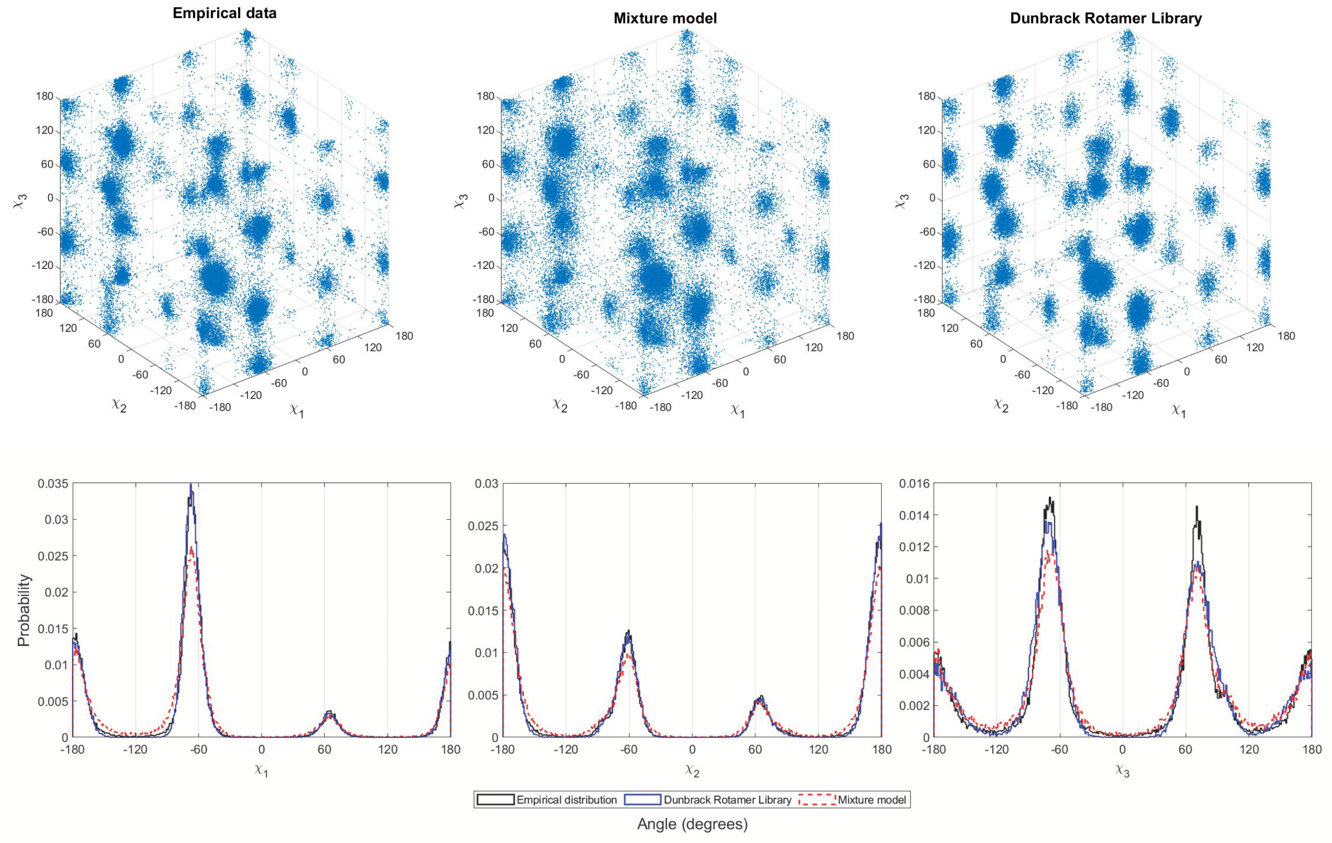

(a) Methionine (MET)

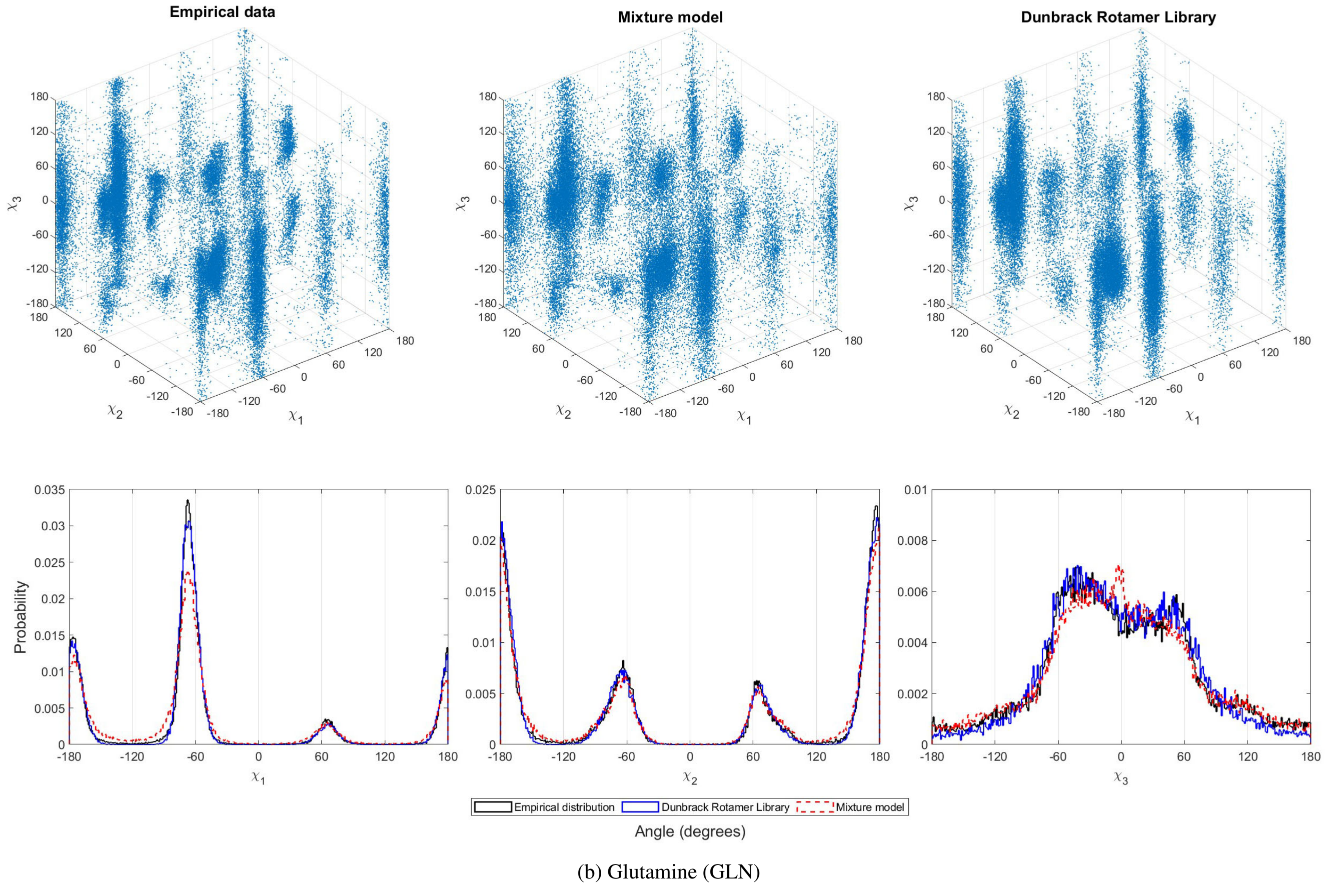

(b) Glutamine (GLN)

Figure SF 2. (a) The projection, into the sidechain (χ_1, χ_2, χ_3) space (unwrapped), of 50,000 randomly sampled points (vector of dihedral angles) for the amino acid Methionine (MET) from MML mixture model (first row, center), of the same number of points from the Dunbrack model (first row, right), and of the observed (empirical) distribution of the same angles (first row, left) from PDBHighRes. In the plots of the second row, the same data is visualized differently over three separate plots, with each of the three sidechain dihedral angles as x-axis (unwrapped), with y-axis showing the corresponding relative probabilities (in a 1° intervals). (b) The third and fourth rows plots are similar to first and second, respectively, but for the non-rotameric amino acid, Glutamine (GLN).

S7 Qualitative comparison of model fit across all amino acid sidechain dihedral angles for PDB50HighRes

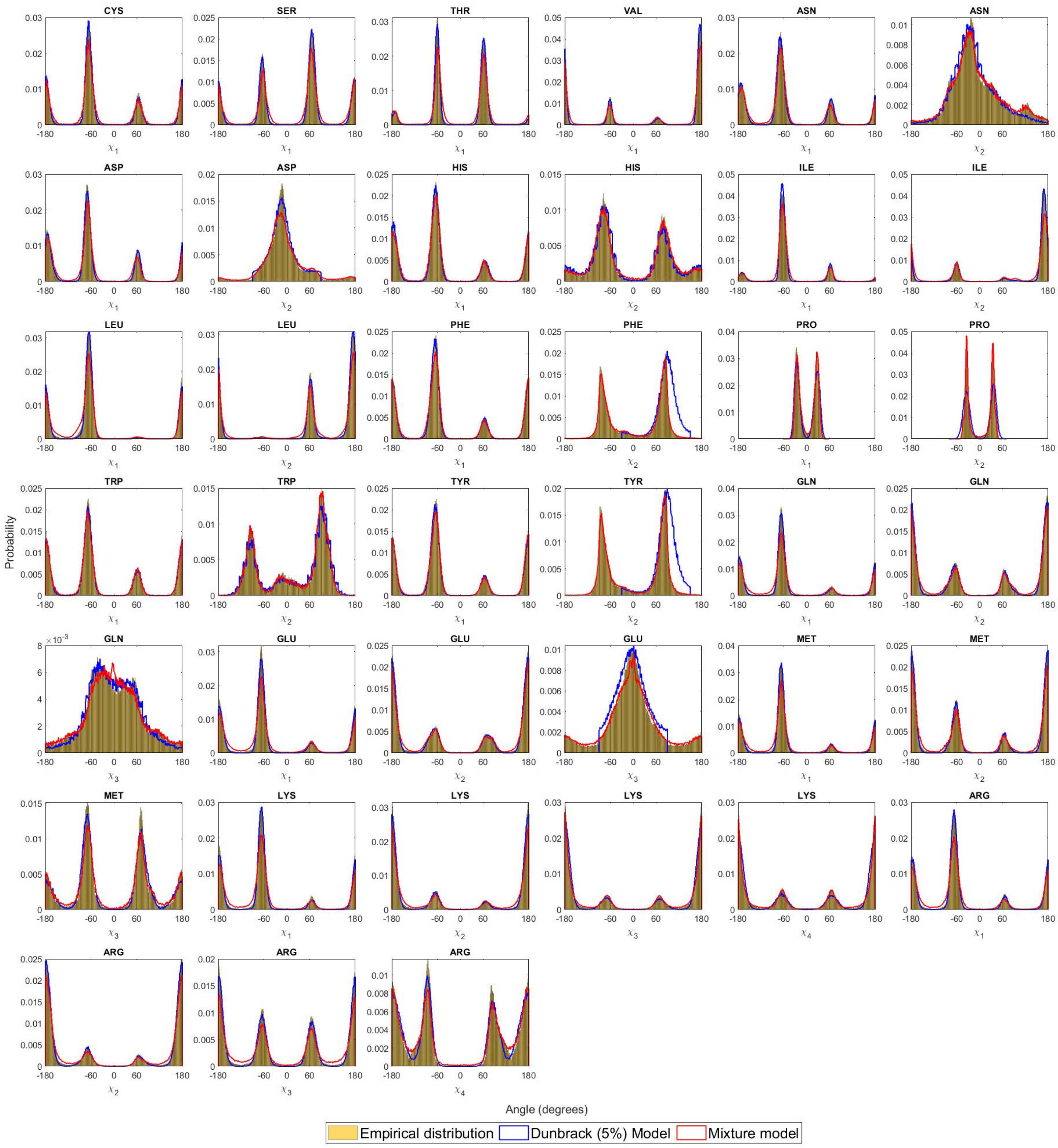

Figure SF 3. Fidelity of the inferred MML mixure models: the projected distribution of individual sidechain dihedral angles across all amino acids derived by randomly sampling $N^{(aa)}$ datapoints (see Table ST 2) from MML-derived mixture models and Dunbrack (5% smoothed) library, and compared to the empirical distribution.

S8 KL-divergence between mixture models inferred on PDB50 and PDB50HighRes dataset

Kullback-Leibler (KL) divergence informs a measure of relative entropy needed for encoding data over a probability model relative to another. We used this measure to evaluate mixture models derived for PDB50 and PDB50HighRes datasets under an empirical method to estimate KL divergence. Consider a set of data points $x_i; \forall 1 \leq i \leq N$ sampled from a true mixture distribution $\mathcal{M}^t$. Then the *empirical* KL-divergence $D_{KL}(\mathcal{M}^t \parallel \mathcal{M}))$ between $\mathcal{M}^t$ (true distribution) and another mixture model $\mathcal{M}$ can be approximated as (Kasarapu and Allison, 2015),

$$D_{KL}(\mathcal{M}^t \parallel \mathcal{M}) = E_{\mathcal{M}^t}\left[\log\left(\frac{\Pr(x_i;\mathcal{M}^t)}{\Pr(x_i;\mathcal{M})}\right)\right] \approx \frac{1}{N}\sum_{i=1}^{N}\log\left(\frac{\Pr(x_i;\mathcal{M}^t)}{\Pr(x_i;\mathcal{M})}\right) \tag{10}$$

Table ST 4 shows KL-divergence between the mixture models inferred from the two datasets. The low KL-divergence values provide evidence that PDB50 and PDB50HighRes models are practically similar to sample representative amino acid conformations in proteins.

Table ST 4. This table illustrates the KL-divergence between mixture models inferred from PDB50 and PDB50HighRes datasets for each amino acid (a.a)

a.a	KL-divergence	a.a	KL-divergence
ALA	0.02675	MET	0.31734
CYS	0.05314	ASN	0.15406
ASP	0.26640	PRO	0.42636
GLU	0.20948	GLN	0.24193
PHE	0.16896	ARG	0.18094
GLY	0.03034	SER	0.06501
HIS	0.15723	THR	0.05209
ILE	0.62360	VAL	0.03940
LYS	0.20449	TRP	0.15949
LEU	0.22799	TYR	0.06479

S9 Message length of Dunbrack backbone-dependant rotamer library

For a fair and objective comparison in terms of Shannon information content, we need to translate any Dunbrack model ($\mathcal{D}^{aa}_{rotamer}$) to estimate their equivalent first part and second part message length terms. This is achieved as follows. Dunbrack report their latest backbone-dependent libraries in $10° \times 10°$ bins of $\langle\phi,\psi\rangle$ values. In each bin, they report a statistical model with a fixed number of components determined by the number of discrete rotamer states of the residue being considered. For example, consider the rotamer library for methionine amino acid. Methionine has 3 sidechain angles and each angle is considered to have 3 distinct rotameric states, yielding a total number of $3 \times 3 \times 3 = 27$ possibilities over all the three angles. Hence each bin of the backbone grid of methionine can be directly interpreted as a mixture model containing 27 components. For consistency, we followed the rotamer categorization used in their reported work describing latest libraries (Shapovalov and Dunbrack Jr, 2011). For example, Proline has only 2 rotameric states for χ_1.

Each component of this bin-wise mixture model is considered a product of von Mises distributions (since they independently model each dihedral angle by a von Mises circular distribution) where the weight parameter of each component is their defined conditional probability of each discrete rotameric state. Specifically, each component of a selected bin-wise mixture of methionine will be a product of 3 von Mises distributions where the parameters of each von Mises distribution are directly mapped from the parameters they report in their library.

Consider a d-dimensional dihedral angle datum (backbone + sidechain) $x_i \in X$ where X denotes the input set of N observations from a non-redundant protein dataset. Under this bin-wise mixture representation, we compared the complexity and fidelity of MML-derived mixture models and the Dunbrack rotamer library for stating N observations in 2 possible ways.

1. The backbone dihedral angles ϕ and ψ under the Dunbrack model are stated over a uniform distribution.
2. For each MML-inferred mixture model drop/ignore the von Mises terms corresponding to backbone dihedral angles when calculating the second part.

Approach 1

Calculating second part. We first select the bin in the Dunbrack model into which any specfic ϕ_i and ψ_i of x_i falls. Then the mixture model of sidechain dihedral angles specified by the $\langle\phi_i,\psi_i\rangle$-bin is used to encode the sidechain angles observed for x_i, identical to how we encode the same using the MML model. For the methionine example, the message length associated with stating sidechain dihedral angles of x_i is,

$$I(\langle x_{i_3}, x_{i_4}, x_{i_5}\rangle\,|\mathcal{D}^{\text{MET}}_{\text{rotamer}}) = -\log\left(\sum_{j=1}^{27}\left(w_j\prod_{p=3}^{5} f(x_{i_p}|\,\langle\mu_{j_p},\kappa_{j_p}\rangle)\right)\epsilon^3\right) \tag{11}$$

Here mixture component, $\prod_{p=3}^{5} f(x_{i_p}|\,\langle\mu_{j_p},\kappa_{j_p}\rangle)$ denote the jth rotameric state of $x_{i_3}, x_{i_4}, x_{i_5}$ angles in ϕ_i,ψ_i bin. $f(x_{i_p}|\,\langle\mu_{j_p},\kappa_{j_p}\rangle$ represent von Mises distribution of sidechain dihedral angle x_{i_p} with parameters μ_{j_p}(mean) and κ_{j_p} (concentration). As with our models, the ϵ is set to 0.0873 radians (see main text for details).

In the first approach, we state the backbone dihedral angles of x_i under a uniform distribution. Hence the message length of nominating a $10° \times 10°$ $\langle\phi,\psi\rangle$-bin losslessly takes $\log(36^2) = 2 \times \log 36$ bits (assume logarithms are all base-2). Further any statement of the observed ϕ and ψ uniformly distributed within each $10°$ interval defines a probability of $\frac{\epsilon°}{10°}$, and negative logarithm of that probability yields $\log(10°/\epsilon°)$ bits. Thus, the amount of information to state the two backbone dihedrals under this approach losslessly takes $2 \times (\log 36 + \log(10°/\epsilon°))$ bits. Hence the second part of stating datum x_i of methionine under $\mathcal{D}^{\text{MET}}_{\text{rotamer}}$ can be quantified as

$$I\left(x_i|\mathcal{D}^{\text{MET}}_{\text{rotamer}}\right) = I(\langle x_{i_3}, x_{i_4}, x_{i_5}\rangle\,|\mathcal{D}^{\text{MET}}_{\text{rotamer}}) + 2(\log(36) + \log(10°/\epsilon°)). \tag{12}$$

Hence, the total message length of encoding N observations is the summation of individual message length terms $I\left(x_i|\mathcal{D}^{\text{MET}}_{\text{rotamer}}\right), \forall \leq i \leq N$

Calculating first part. The first part term of the $\mathcal{D}^{\text{aa}}_{\text{rotamer}}$ contains the message length of stating all the mixture parameters (across $36^2 = 1,296$) bins and the message length of stating the backbone dihedral angle parameters. For a single ϕ,ψ bin, the first part term of the mixture model's message length is the summation of 3 message length terms (see Section 2.3 in the main text) associated with stating mixture components, weights of the mixture and component parameters. We calculate these three terms using exactly the same method we use to calculate the MML mixture model's first part (as described in the main text). The total message of stating all mixture parameters is calculated by summing the first part terms over $1,296$ mixtures.

Approach 2

In the second approach, we entirely ignore stating the backbone dihedral angles, by ignoring the corresponding von Mises terms from the derived mixture models and this allows us to compare with Dunbrack's models on an equal footing to explain only sidechain dihedral angles, which they are geared to explain.

Calculating second part. Similar to the first approach, we calculate the message length of stating sidechain dihedral angles of a d-dimensional datum (x_i) by selecting the mixture model associated with the bin into which any observed ϕ_i,ψ_i falls, and using that implied mixture model in the Dunbrack's library to determine the message length of stating sidechain angles of x_i. Compared to the first approach, in this method, we are not sending the backbone dihedral angles. But for Dunbrack's model, we still need to include the corresponding bin information in the second part of the message so that a receiver is able to recover the data losslessly by selecting the correct mixture model. Without that information, the message will not be lossless. Hence, the message length of stating a backbone bin is simply the information content associated with selecting a bin out of 1296 possible bins (assuming each bin is equally probable). As before, as in illustrative example we can apply this to the methionine example to calculate the message length of x_i,

$$I(\langle x_{i_3}, x_{i_4}, x_{i_5}\rangle \,|\mathcal{D}^{\text{MET}}_{\text{rotamer}}) = -\log\left(\sum_{j=1}^{27}\left(w_j \prod_{p=3}^{5} f(x_{i_p}\,|\,\langle\mu_{j_p}, \kappa_{j_p}\rangle)\right)\epsilon^3\right) + \log(1296) \quad (13)$$

Equation 13 states the message length of stating a single datum only. The message length of stating all the data can be calculated by summing over individual message length terms $I\left(\langle x_{i_3}, x_{i_4}, x_{i_5}\rangle \,|\mathcal{D}^{\text{MET}}_{\text{rotamer}}\right), \forall \leq i \leq N$.

Calculating first part. Similar to calculating the first-part term in the first approach, we calculate the message length required to state parameters of 1296 mixture models in Dunbrack's library, and on the MML side, we ignore the message length associated with stating the parameters associated with backbone dihedral angles.

S10 Sampling from PhiSiCal mixture models

The collection of mixture models and conformation sampling methods are accessible from http://lcb.infotech.monash.edu.au/phisical. The formal statistical distributions provide direct ways to sample under their implied distributions. Specifically, each mixture model reports a set of weight parameters of mixture components (product of von Mises distributions) along with their associated parameter estimates. For individual amino acids, the inferred mixture model can be used to randomly sample its dihedral angles in two distinct ways: (1) jointly sample backbone and sidechain dihedral angles $\langle \phi, \psi, \chi_1, \chi_2, ...\rangle$, and (2) conditionally sample only sidechain dihedral angles $\langle \chi_1, \chi_2, ...\rangle$ given any specified backbone dihedral angles $\langle \phi, \psi\rangle$.

Sampling joint (mainchain and sidechain) dihedral angles

Sampling any (backbone and sidechain) dihedral angle vector for any specified amino acid involves these operations:

1. First, identify the inferred PhiSiCal mixture model associated with the specified amino acid.
2. Probabilistically select a component from that identified mixture model. That is, randomly select a component based on the inferred weight parameters of the mixture model. These weights give the probability of selecting a component in the mixture.
3. Once a component is identified, for each von Mises term (in the product of such terms) of that component, randomly sample a dihedral angle from that von Mises distribution given its (μ, κ) parameters.

The resultant dihedral angle vector is a sample from the joint distribution.

Sampling sidechain dihedral angles conditional on backbone dihedral angles

Importantly, given the Bayesian framework on which these models stand, a simple technique of *posterior reweighting* can be employed on any of the inferred mixture models to transform them into conditional distributions, and sample conditionally sidechain dihedrals given any specified backbone dihedral angles. In this method of posterior reweighting, for a mixture model $\mathcal{M}(\Lambda) = \sum_{j=1}^{|\mathcal{M}|} w_i f_i(\Theta_j)$ (see Equation 1 in the main text), only its component-weights $w_1, w_2, \ldots w_{|\mathcal{M}|}$ are updated to $w'_1, w'_2, \ldots w'_{|\mathcal{M}|}$, such that each w'_i is the corresponding posterior probability of the component given $\langle \phi, \psi\rangle$. (Note, the other parameters of the original mixture model are left intact and only the original inferred weights of the mixture model are updated). The resultant $\mathcal{M}(\Lambda|\,\langle \phi, \psi\rangle) = \sum_{j=1}^{|\mathcal{M}|} w'_i f_i(\Theta_j)$ is now a conditional mixture model (given any $\langle \phi, \psi\rangle$). Since this is yet another mixture model, it can be sampled using the same approach as the one described in the section above. The elegance of this approach is that only the originally inferred 'beliefs' of the component probabilities (i.e., the component weights as given by the original joint mixture model) have been updated based on evidence of some observed $\langle \phi, \psi\rangle$, thus yielding a mixture model conditioned on those observations to sample from.

S11 Assessing the stability of the search algorithm

The employed search algorithm is a core component of our inference process to determine the optimal mixture model *explaining* underlying dihedral angle distributions *unsupervised*. Hence we evaluated the stability of the solutions of this search process thoroughly to assess its applicability to this modelling challenge. The following discusses the method that we employed to evaluate the validity of the solutions.

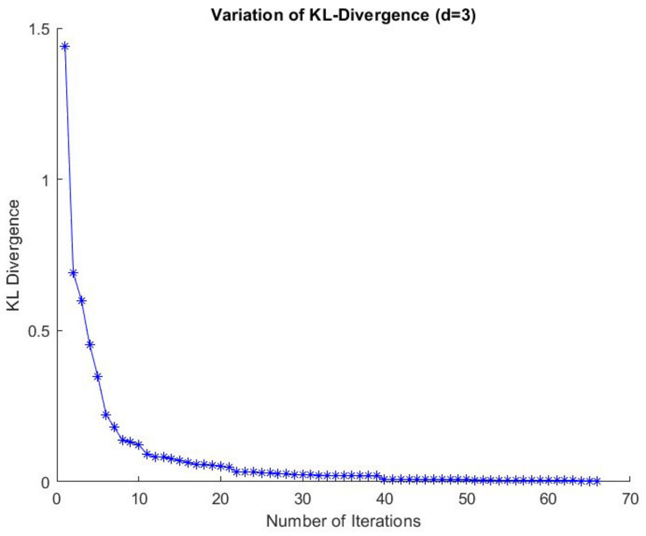

Figure SF 4. Variation of KL-divergence of a mixture inferred from a 3-dimension dihedral angle dataset. During 66 iterations this mixture settled in to 66 components.

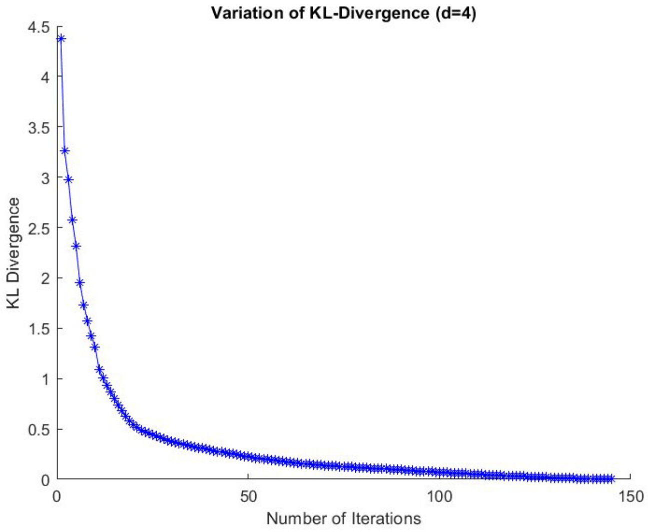

Figure SF 5. Variation of KL-divergence of a mixture inferred from a 4-dimension dihedral angle dataset. During 145 iterations this mixture settled in to 143 components.

S11.0.1 Method

For this task, synthetic data was generated by randomly sampling from mixture models with component parameters and the number of components known a priori. Then the sampled data was utilized to infer new mixture models. In order to assess the reliability of the underlying search process, the derived new mixture models were compared against their original mixture models (true distributions), under the KL-divergence metric (see Equation 10). We considered original mixture distributions having similar dimensions observed in amino acid dihedral angles ($d = 2, .., 7$) and having number of components between 20 - 300 range.

Figures SF 4, SF 5 and SF 6 show variation of KL-divergence between original distribution and inferred model during different iterations of the search process. Starting from a single component and increase(decrease) component count from split(merge,delete) operations and until convergence. From these illustrations it is clearly evident that employed search process converges *near* to the ground truth.

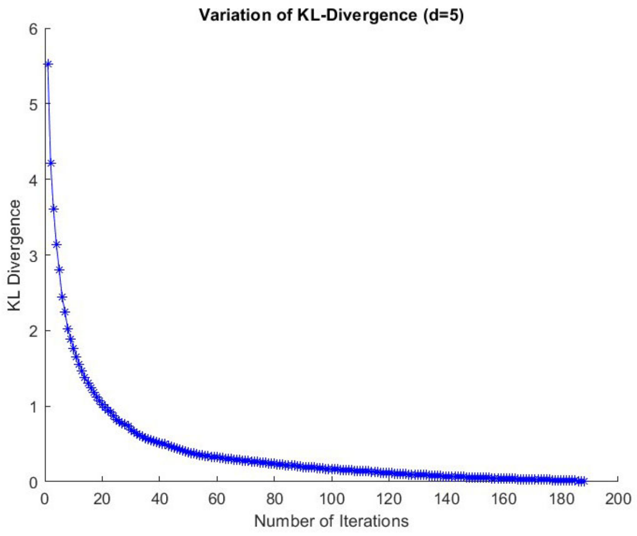

Figure SF 6. Variation of KL-divergence of a mixture inferred from a 5-dimension dihedral angle dataset. During 188 iterations this mixture settled in to 180 components.

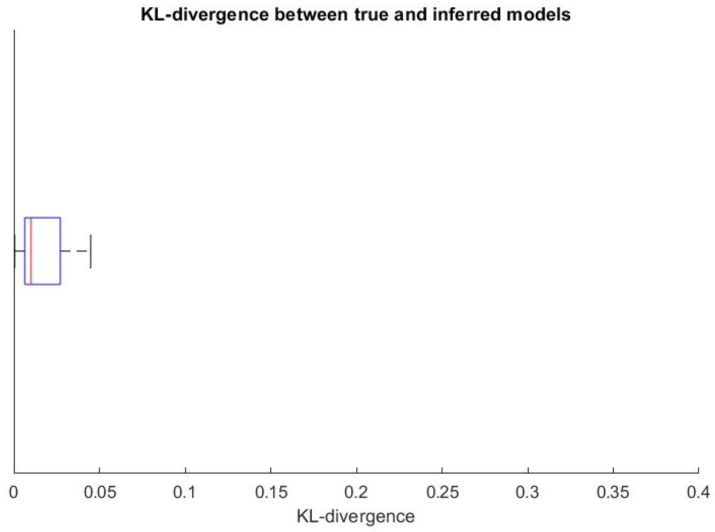

Figure SF 7. Quartile statistics of KL-divergence between true and inferred distributions (converged) from 17 experiments.

Further we observe at convergence KL-divergence quartile statistics, Q_1=0.0076, Q_2 = 0.0100 and Q_3=0.0259 from 17 experiments with varying number of components and dihedral angle dimensions (see Figure SF 7). These statistics further support the stability of the solutions of the underlying EM search process (true and the inferred models are *nearly* similar).

S12 Comparing different smoothing levels of Dunbrack's rotamer library.

In the main text, we presented the comparison between MML-derived mixture models and the Dunbrack rotamer library at 5% smoothing, which were the default libraries that Shapovalov and Dunbrack Jr (2011) earmarked as the best performing. To complete the comparison, we present below additional tables of comparison between the MML-derived mixture models and Dunbrack's rotamer library at varying (2%, 10%, 20%, and 25%) smoothing levels. See supplementary tables ST 5, ST 6, ST 7, ST 8 below.
Further, Figures SF 8, SF 9, SF 10 and SF 11, show a qualitative comparison between the MML-derived mixture model against the Dunbrack's library at those different smoothing levels.
These tables and figures clearly illustrate that the accuracy of Dunbrack's models starts to decline when the smoothing level increases. For example, one can see the decline in the accuracy of the χ_2 of TRP, χ_3 of GLU, χ_3 of GLN when smoothing value increases from 2% to 25% (see Figures SF 8 to SF 11).

S12.1 Message lengths for stating amino acid sidechain dihedral angles from PDB50 dataset: Quantitative comparison for 2% smoothing level of Dunbrack library

Table ST 5. This table illustrates a quantitative comparison between the MML-inferred mixture model ($\mathcal{M}^{(\text{aa})}$) and that of Dunbrack rotamer library ($\mathcal{D}^{(\text{aa})}_{\text{rotamer}}$) with 2% smoothing level to state sidechain dihedral angles of each of the twenty naturally occurring amino acids (aa). Here we have only considered the cost of stating the sidechain dihedral angles of each of the twenty naturally occurring amino acids (aa) and omitted the backbone $\langle \phi, \psi \rangle$. The 'N/A' terms across Alanine (ALA) and Glycine (GLY) arise because those amino acids do not have sidechain dihedral angles.

		MML Mixture Model ($\mathcal{M}^{(\text{aa})}$) message length statistics in bits (rounded)					Dunbrack Rotamer Library ($\mathcal{D}^{(\text{aa})}_{\text{rotamer}}$) message length statistics in bits (rounded)					Null Model (Raw) in bits	
(aa)	$N^{(\text{aa})}$	($\lvert\mathcal{M}^{(\text{aa})}\rvert$, $\lvert\Lambda^{(\text{aa})}\rvert$)	first-part (complexity)	second-part (fit)	Total (complexity+fit)	$\frac{\text{Total}}{N^{(\text{aa})}}$	($\lvert\mathcal{D}^{(\text{aa})}_{\text{rotamer}}\rvert$; #Params)	first-part (complexity)	second-part (fit)	Total (complexity+fit)	$\frac{\text{Total}}{N^{(\text{aa})}}$	Null($X^{(\text{aa})}$)	$\frac{\text{Null}(X^{(\text{aa})})}{N^{(\text{aa})}}$
LEU	2,171,630	(165;1,484)	4,095	17,578,246	17,582,342	8.1	(11,664;57,024)	1,038,297	41,823,773	42,862,070	19.7	26,797,588	12.3
ALA	1,861,359	(25;124)	N/A	N/A	N/A	N/A	(N/A;N/A)	N/A	N/A	N/A	N/A	N/A	N/A
VAL	1,601,058	(96;671)	1,625	6,607,950	6,609,575	4.1	(3,888;10,368)	217,597	23,549,959	23,767,556	14.8	9,878,408	6.2
GLY	1,588,115	(30;149)	N/A	N/A	N/A	N/A	(N/A;N/A)	N/A	N/A	N/A	N/A	N/A	N/A
GLU	1,446,860	(262;2,881)	7,754	21,695,912	21,703,666	15.0	(69,984;488,592)	9,557,315	37,051,108	46,608,423	32.2	26,781,053	18.5
SER	1,337,273	(114;797)	1,757	6,592,674	6,594,431	4.9	(3,888;10,368)	210,873	20,961,278	21,172,151	15.8	8,250,874	6.2
ILE	1,333,508	(172;1,547)	4,346	10,688,100	10,692,445	8.0	(11,664;57,024)	929,222	25,042,446	25,971,668	19.5	16,455,289	12.3
ASP	1,279,567	(170;1,529)	3,640	12,462,677	12,466,317	9.7	(23,328;115,344)	2,340,479	25,105,109	27,445,589	21.4	15,789,665	12.3
THR	1,221,604	(90;629)	1,445	5,531,460	5,532,905	4.5	(3,888;10,368)	212,264	18,316,720	18,528,984	15.2	7,537,205	6.2
LYS	1,176,395	(266;3,457)	9,833	22,078,096	22,087,929	18.8	(104,976;943,488)	13,524,209	35,501,351	49,025,560	41.7	29,033,076	24.7
ARG	1,130,448	(250;3,749)	12,164	23,504,690	23,516,854	20.8	(104,976;943,488)	14,637,302	35,020,688	49,657,990	43.9	34,873,897	30.8
PRO	1,004,859	(231;2,078)	8,956	5,257,024	5,265,980	5.2	(2,592;11,664)	255,399	16,318,837	16,574,237	16.5	12,399,809	12.3
ASN	948,274	(180;1,619)	3,703	9,582,207	9,585,910	10.1	(46,656;231,984)	4,513,404	19,373,159	23,886,563	25.2	11,701,559	12.3
PHE	927,298	(226;2,033)	5,401	8,460,779	8,466,181	9.1	(23,328;115,344)	2,168,432	17,228,831	19,397,263	20.9	11,442,718	12.3
GLN	820,871	(239;2,628)	6,804	12,270,999	12,277,803	15.0	(139,968;978,480)	17,500,129	21,548,829	39,048,958	47.6	15,194,138	18.5
TYR	788,176	(192;1,727)	4,480	7,193,098	7,197,578	9.1	(23,328;115,344)	2,234,390	14,604,830	16,839,220	21.4	9,725,974	12.3
HIS	515,611	(163;1,466)	3,443	5,175,762	5,179,204	10.0	(46,656;231,984)	4,317,912	10,395,540	14,713,451	28.5	6,362,562	12.3
MET	417,170	(270;2,969)	7,919	5,954,095	5,962,013	14.3	(34,992;243,648)	4,114,107	10,682,963	14,797,070	35.5	7,721,723	18.5
TRP	310,470	(212;1,907)	4,816	2,958,040	2,962,856	9.5	(46,656;231,984)	3,981,481	6,031,196	10,012,677	32.3	3,831,153	12.3
CYS	296,547	(96;671)	1,433	1,389,186	1,390,619	4.7	(3,888;10,368)	191,072	4,438,901	4,629,973	15.6	1,829,673	6.2

S12.2 Message lengths for stating amino acid sidechain dihedral angles from PDB50 dataset: Quantitative comparison for 10% smoothing level of Dunbrack library

Table ST 6. This table illustrates a quantitative comparison between the MML-inferred mixture model ($\mathcal{M}^{(aa)}$) and that of Dunbrack rotamer library ($\mathcal{D}^{(aa)}_{\text{rotamer}}$) with 10% smoothing level to state sidechain dihedral angles of each of the twenty naturally occurring amino acids (aa). Here we have only considered the cost of stating the sidechain dihedral angles of each of the twenty naturally occurring amino acids (aa) and omitted the backbone $\langle\phi,\psi\rangle$. The 'N/A' terms across Alanine (ALA) and Glycine (GLY) arise because those amino acids do not have sidechain dihedral angles.

		MML Mixture Model ($\mathcal{M}^{(aa)}$) message length statistics in bits (rounded)					Dunbrack Rotamer Library ($\mathcal{D}^{(aa)}_{\text{rotamer}}$) message length statistics in bits (rounded)					Null Model (Raw) in bits	
(aa)	$N^{(aa)}$	($\lvert\mathcal{M}^{(aa)}\rvert$, $\lvert\Lambda^{(aa)}\rvert$)	first-part (complexity)	second-part (fit)	Total (complexity+fit)	$\frac{\text{Total}}{N^{(aa)}}$	($\lvert\mathcal{D}^{(aa)}_{\text{rotamer}}\rvert$; #Params)	first-part (complexity)	second-part (fit)	Total (complexity+fit)	$\frac{\text{Total}}{N^{(aa)}}$	Null($X^{(aa)}$)	$\frac{\text{Null}(X^{(aa)})}{N^{(aa)}}$
LEU	2,171,630	(165;1,484)	4,095	17,578,246	17,582,342	8.1	(11,664;57,024)	1,137,226	41,710,760	42,847,985	19.7	26,797,588	12.3
ALA	1,861,359	(25;124)	N/A	N/A	N/A	N/A	(N/A;N/A)	N/A	N/A	N/A	N/A	N/A	N/A
VAL	1,601,058	(96;671)	1,625	6,607,950	6,609,575	4.1	(3,888;10,368)	216,601	23,551,213	23,767,814	14.8	9,878,408	6.2
GLY	1,588,115	(30;149)	N/A	N/A	N/A	N/A	(N/A;N/A)	N/A	N/A	N/A	N/A	N/A	N/A
GLU	1,446,860	(262;2,881)	7,754	21,695,912	21,703,666	15.0	(69,984;488,592)	9,902,176	37,023,602	46,925,778	32.4	26,781,053	18.5
SER	1,337,273	(114;797)	1,757	6,592,674	6,594,431	4.9	(3,888;10,368)	210,474	20,922,682	21,133,156	15.8	8,250,874	6.2
ILE	1,333,508	(172;1,547)	4,346	10,688,100	10,692,445	8.0	(11,664;57,024)	1,011,840	24,987,375	25,999,216	19.5	16,455,289	12.3
ASP	1,279,567	(170;1,529)	3,640	12,462,677	12,466,317	9.7	(23,328;115,344)	2,334,307	25,077,909	27,412,216	21.4	15,789,665	12.3
THR	1,221,604	(90;629)	1,445	5,531,460	5,532,905	4.5	(3,888;10,368)	211,193	18,261,872	18,473,065	15.1	7,537,205	6.2
LYS	1,176,395	(266;3,457)	9,833	22,078,096	22,087,929	18.8	(104,976;943,488)	14,982,586	35,432,372	50,414,958	42.9	29,033,076	24.7
ARG	1,130,448	(250;3,749)	12,164	23,504,690	23,516,854	20.8	(104,976;943,488)	16,182,120	34,966,919	51,149,039	45.2	34,873,897	30.8
PRO	1,004,859	(231;2,078)	8,956	5,257,024	5,265,980	5.2	(2,592;11,664)	254,275	16,307,481	16,561,756	16.5	12,399,809	12.3
ASN	948,274	(180;1,619)	3,703	9,582,207	9,585,910	10.1	(46,656;231,984)	4,590,827	19,367,626	23,958,453	25.3	11,701,559	12.3
PHE	927,298	(226;2,033)	5,401	8,460,779	8,466,181	9.1	(23,328;115,344)	2,296,537	17,252,420	19,548,957	21.1	11,442,718	12.3
GLN	820,871	(239;2,628)	6,804	12,270,999	12,277,803	15.0	(139,968;978,480)	19,052,891	21,526,082	40,578,973	49.4	15,194,138	18.5
TYR	788,176	(192;1,727)	4,480	7,193,098	7,197,578	9.1	(23,328;115,344)	2,260,947	14,620,212	16,881,159	21.4	9,725,974	12.3
HIS	515,611	(163;1,466)	3,443	5,175,762	5,179,204	10.0	(46,656;231,984)	4,384,777	10,386,932	14,771,709	28.6	6,362,562	12.3
MET	417,170	(270;2,969)	7,919	5,954,095	5,962,013	14.3	(34,992;243,648)	4,336,874	10,655,090	14,991,964	35.9	7,721,723	18.5
TRP	310,470	(212;1,907)	4,816	2,958,040	2,962,856	9.5	(46,656;231,984)	4,148,829	6,055,285	10,204,114	32.9	3,831,153	12.3
CYS	296,547	(96;671)	1,433	1,389,186	1,390,619	4.7	(3,888;10,368)	189,723	4,428,936	4,618,660	15.6	1,829,673	6.2

S12.3 Message lengths for stating amino acid sidechain dihedral angles from PDB50 dataset: Quantitative comparison for 20% smoothing level of Dunbrack library

Table ST 7. This table illustrates a quantitative comparison between the MML-inferred mixture model ($\mathcal{M}^{(aa)}$) and that of Dunbrack rotamer library ($\mathcal{D}^{(aa)}_{\text{rotamer}}$) with 20% smoothing level to state sidechain dihedral angles of each of the twenty naturally occurring amino acids (aa). Here we have only considered the cost of stating the sidechain dihedral angles of each of the twenty naturally occurring amino acids (aa) and omitted the backbone $\langle\phi,\psi\rangle$. The 'N/A' terms across Alanine (ALA) and Glycine (GLY) arise because those amino acids do not have sidechain dihedral angles.

		MML Mixture Model ($\mathcal{M}^{(aa)}$) message length statistics in bits (rounded)					Dunbrack Rotamer Library ($\mathcal{D}^{(aa)}_{\text{rotamer}}$) message length statistics in bits (rounded)					Null Model (Raw) in bits	
(aa)	$N^{(aa)}$	($\lvert\mathcal{M}^{(aa)}\rvert$, $\lvert\Lambda^{(aa)}\rvert$)	first-part (complexity)	second-part (fit)	Total (complexity+fit)	$\frac{\text{Total}}{N^{(aa)}}$	($\lvert\mathcal{D}^{(aa)}_{\text{rotamer}}\rvert$; #Params)	first-part (complexity)	second-part (fit)	Total (complexity+fit)	$\frac{\text{Total}}{N^{(aa)}}$	Null($X^{(aa)}$)	$\frac{\text{Null}(X^{(aa)})}{N^{(aa)}}$
LEU	2,171,630	(165;1,484)	4,095	17,578,246	17,582,342	8.1	(11,664;57,024)	1,204,083	41,623,038	42,827,121	19.7	26,797,588	12.3
ALA	1,861,359	(25;124)	N/A	N/A	N/A	N/A	(N/A;N/A)	N/A	N/A	N/A	N/A	N/A	N/A
VAL	1,601,058	(96;671)	1,625	6,607,950	6,609,575	4.1	(3,888;10,368)	215,569	23,595,581	23,811,150	14.9	9,878,408	6.2
GLY	1,588,115	(30;149)	N/A	N/A	N/A	N/A	(N/A;N/A)	N/A	N/A	N/A	N/A	N/A	N/A
GLU	1,446,860	(262;2,881)	7,754	21,695,912	21,703,666	15.0	(69,984;488,592)	10,109,636	37,022,486	47,132,122	32.6	26,781,053	18.5
SER	1,337,273	(114;797)	1,757	6,592,674	6,594,431	4.9	(3,888;10,368)	210,388	20,902,387	21,112,775	15.8	8,250,874	6.2
ILE	1,333,508	(172;1,547)	4,346	10,688,100	10,692,445	8.0	(11,664;57,024)	1,119,018	24,951,525	26,070,543	19.6	16,455,289	12.3
ASP	1,279,567	(170;1,529)	3,640	12,462,677	12,466,317	9.7	(23,328;115,344)	2,325,771	25,168,174	27,493,945	21.5	15,789,665	12.3
THR	1,221,604	(90;629)	1,445	5,531,460	5,532,905	4.5	(3,888;10,368)	211,124	18,273,176	18,484,300	15.1	7,537,205	6.2
LYS	1,176,395	(266;3,457)	9,833	22,078,096	22,087,929	18.8	(104,976;943,488)	15,458,386	35,415,191	50,873,578	43.2	29,033,076	24.7
ARG	1,130,448	(250;3,749)	12,164	23,504,690	23,516,854	20.8	(104,976;943,488)	16,740,256	34,949,127	51,689,383	45.7	34,873,897	30.8
PRO	1,004,859	(231;2,078)	8,956	5,257,024	5,265,980	5.2	(2,592;11,664)	253,869	16,319,192	16,573,062	16.5	12,399,809	12.3
ASN	948,274	(180;1,619)	3,703	9,582,207	9,585,910	10.1	(46,656;231,984)	4,585,079	19,443,062	24,028,141	25.3	11,701,559	12.3
PHE	927,298	(226;2,033)	5,401	8,460,779	8,466,181	9.1	(23,328;115,344)	2,290,175	17,377,675	19,667,850	21.2	11,442,718	12.3
GLN	820,871	(239;2,628)	6,804	12,270,999	12,277,803	15.0	(139,968;978,480)	19,446,584	21,542,593	40,989,176	49.9	15,194,138	18.5
TYR	788,176	(192;1,727)	4,480	7,193,098	7,197,578	9.1	(23,328;115,344)	2,253,206	14,733,786	16,986,991	21.6	9,725,974	12.3
HIS	515,611	(163;1,466)	3,443	5,175,762	5,179,204	10.0	(46,656;231,984)	4,374,205	10,421,993	14,796,198	28.7	6,362,562	12.3
MET	417,170	(270;2,969)	7,919	5,954,095	5,962,013	14.3	(34,992;243,648)	4,449,503	10,637,850	15,087,353	36.2	7,721,723	18.5
TRP	310,470	(212;1,907)	4,816	2,958,040	2,962,856	9.5	(46,656;231,984)	4,222,971	6,112,991	10,335,962	33.3	3,831,153	12.3
CYS	296,547	(96;671)	1,433	1,389,186	1,390,619	4.7	(3,888;10,368)	189,461	4,427,876	4,617,337	15.6	1,829,673	6.2

S12.4 Message lengths for stating amino acid sidechain dihedral angles from PDB50 dataset: Quantitative comparison for 25% smoothing level of Dunbrack library

Table ST 8. This table illustrates a quantitative comparison between the MML-inferred mixture model ($\mathcal{M}^{(\text{aa})}$) and that of Dunbrack rotamer library ($\mathcal{D}^{(\text{aa})}_{\text{rotamer}}$) with 25% smoothing level to state sidechain dihedral angles of each of the twenty naturally occurring amino acids (aa). Here we have only considered the cost of stating the sidechain dihedral angles of each of the twenty naturally occurring amino acids (aa) and omitted the backbone $\langle \phi, \psi \rangle$. The 'N/A' terms across Alanine (ALA) and Glycine (GLY) arise because those amino acids do not have sidechain dihedral angles.

		MML Mixture Model ($\mathcal{M}^{(\text{aa})}$) message length statistics in bits (rounded)					Dunbrack Rotamer Library ($\mathcal{D}^{(\text{aa})}_{\text{rotamer}}$) message length statistics in bits (rounded)					Null Model (Raw) in bits	
(aa)	$N^{(\text{aa})}$	$(\lvert\mathcal{M}^{(\text{aa})}\rvert, \lvert\Lambda^{(\text{aa})}\rvert)$	first-part (complexity)	second-part (fit)	Total (complexity+fit)	$\frac{\text{Total}}{N^{(\text{aa})}}$	$(\lvert\mathcal{D}^{(\text{aa})}_{\text{rotamer}}\rvert$; #Params)	first-part (complexity)	second-part (fit)	Total (complexity+fit)	$\frac{\text{Total}}{N^{(\text{aa})}}$	$\text{Null}(X^{(\text{aa})})$	$\frac{\text{Null}(X^{(\text{aa})})}{N^{(\text{aa})}}$
LEU	2,171,630	(165;1,484)	4,095	17,578,246	17,582,342	8.1	(11,664;57,024)	1,202,034	41,619,581	42,821,614	19.7	26,797,588	12.3
ALA	1,861,359	(25;124)	N/A	N/A	N/A	N/A	(N/A;N/A)	N/A	N/A	N/A	N/A	N/A	N/A
VAL	1,601,058	(96;671)	1,625	6,607,950	6,609,575	4.1	(3,888;10,368)	215,428	23,603,397	23,818,825	14.9	9,878,408	6.2
GLY	1,588,115	(30;149)	N/A	N/A	N/A	N/A	(N/A;N/A)	N/A	N/A	N/A	N/A	N/A	N/A
GLU	1,446,860	(262;2,881)	7,754	21,695,912	21,703,666	15.0	(69,984;488,592)	10,117,196	37,040,961	47,158,157	32.6	26,781,053	18.5
SER	1,337,273	(114;797)	1,757	6,592,674	6,594,431	4.9	(3,888;10,368)	210,361	20,901,312	21,111,674	15.8	8,250,874	6.2
ILE	1,333,508	(172;1,547)	4,346	10,688,100	10,692,445	8.0	(11,664;57,024)	1,131,142	24,904,652	26,035,795	19.5	16,455,289	12.3
ASP	1,279,567	(170;1,529)	3,640	12,462,677	12,466,317	9.7	(23,328;115,344)	2,323,875	25,227,990	27,551,865	21.5	15,789,665	12.3
THR	1,221,604	(90;629)	1,445	5,531,460	5,532,905	4.5	(3,888;10,368)	211,013	18,291,157	18,502,169	15.1	7,537,205	6.2
LYS	1,176,395	(266;3,457)	9,833	22,078,096	22,087,929	18.8	(104,976;943,488)	15,751,256	35,410,141	51,161,397	43.5	29,033,076	24.7
ARG	1,130,448	(250;3,749)	12,164	23,504,690	23,516,854	20.8	(104,976;943,488)	17,019,872	34,948,061	51,967,933	46.0	34,873,897	30.8
PRO	1,004,859	(231;2,078)	8,956	5,257,024	5,265,980	5.2	(2,592;11,664)	253,628	16,345,948	16,599,576	16.5	12,399,809	12.3
ASN	948,274	(180;1,619)	3,703	9,582,207	9,585,910	10.1	(46,656;231,984)	4,578,420	19,510,335	24,088,755	25.4	11,701,559	12.3
PHE	927,298	(226;2,033)	5,401	8,460,779	8,466,181	9.1	(23,328;115,344)	2,285,453	17,463,173	19,748,626	21.3	11,442,718	12.3
GLN	820,871	(239;2,628)	6,804	12,270,999	12,277,803	15.0	(139,968;978,480)	19,540,094	21,556,082	41,096,176	50.1	15,194,138	18.5
TYR	788,176	(192;1,727)	4,480	7,193,098	7,197,578	9.1	(23,328;115,344)	2,248,767	14,808,452	17,057,218	21.6	9,725,974	12.3
HIS	515,611	(163;1,466)	3,443	5,175,762	5,179,204	10.0	(46,656;231,984)	4,369,499	10,444,307	14,813,806	28.7	6,362,562	12.3
MET	417,170	(270;2,969)	7,919	5,954,095	5,962,013	14.3	(34,992;243,648)	4,498,337	10,632,880	15,131,217	36.3	7,721,723	18.5
TRP	310,470	(212;1,907)	4,816	2,958,040	2,962,856	9.5	(46,656;231,984)	4,224,051	6,136,965	10,361,015	33.4	3,831,153	12.3
CYS	296,547	(96;671)	1,433	1,389,186	1,390,619	4.7	(3,888;10,368)	189,285	4,429,420	4,618,705	15.6	1,829,673	6.2

S12.5 Model fit across all amino acid sidechain dihedral angles for PDB50 dataset: Qualitative comparison for 2% smoothing level of Dunbrack library

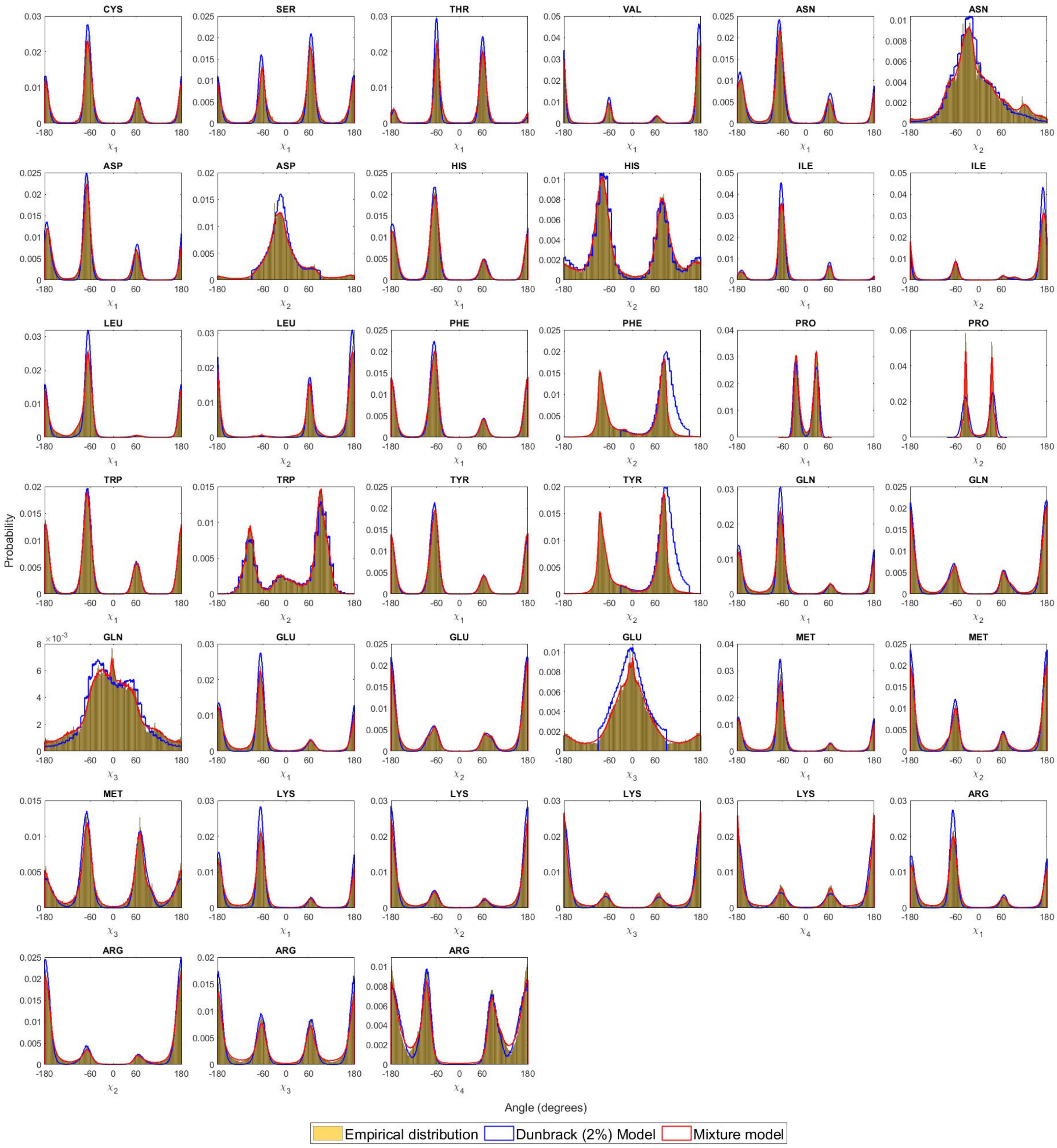

Figure SF 8. Fidelity of the inferred MML mixture models: the projected distribution of individual sidechain dihedral angles across all amino acids derived by randomly sampling N $^{(aa)}$ datapoints (see Table ST 5) from MML-derived mixture models and Dunbrack (2% smoothed) library, and compared to the empirical distribution.

S12.6 Model fit across all amino acid sidechain dihedral angles for PDB50 dataset: Qualitative comparison for 10% smoothing level of Dunbrack library

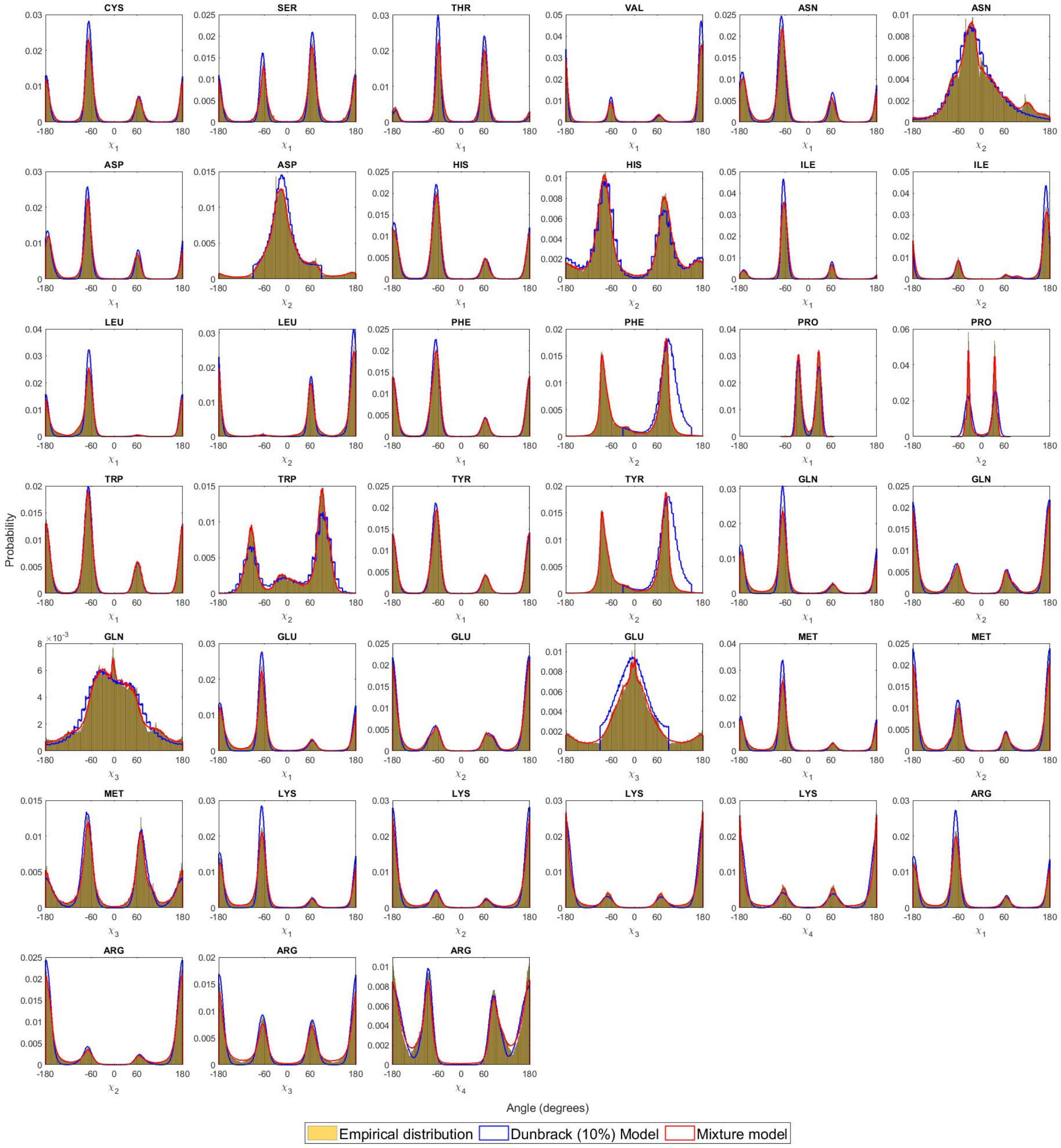

Figure SF 9. Fidelity of the inferred MML mixture models: the projected distribution of individual sidechain dihedral angles across all amino acids derived by randomly sampling N $^{(aa)}$ datapoints (see Table ST 6) from MML derived mixture models and Dunbrack (10% smoothed) library, and compared to the empirical distribution.

S12.7 Model fit across all amino acid sidechain dihedral angles for PDB50 dataset: Qualitative comparison for 20% smoothing level of Dunbrack library

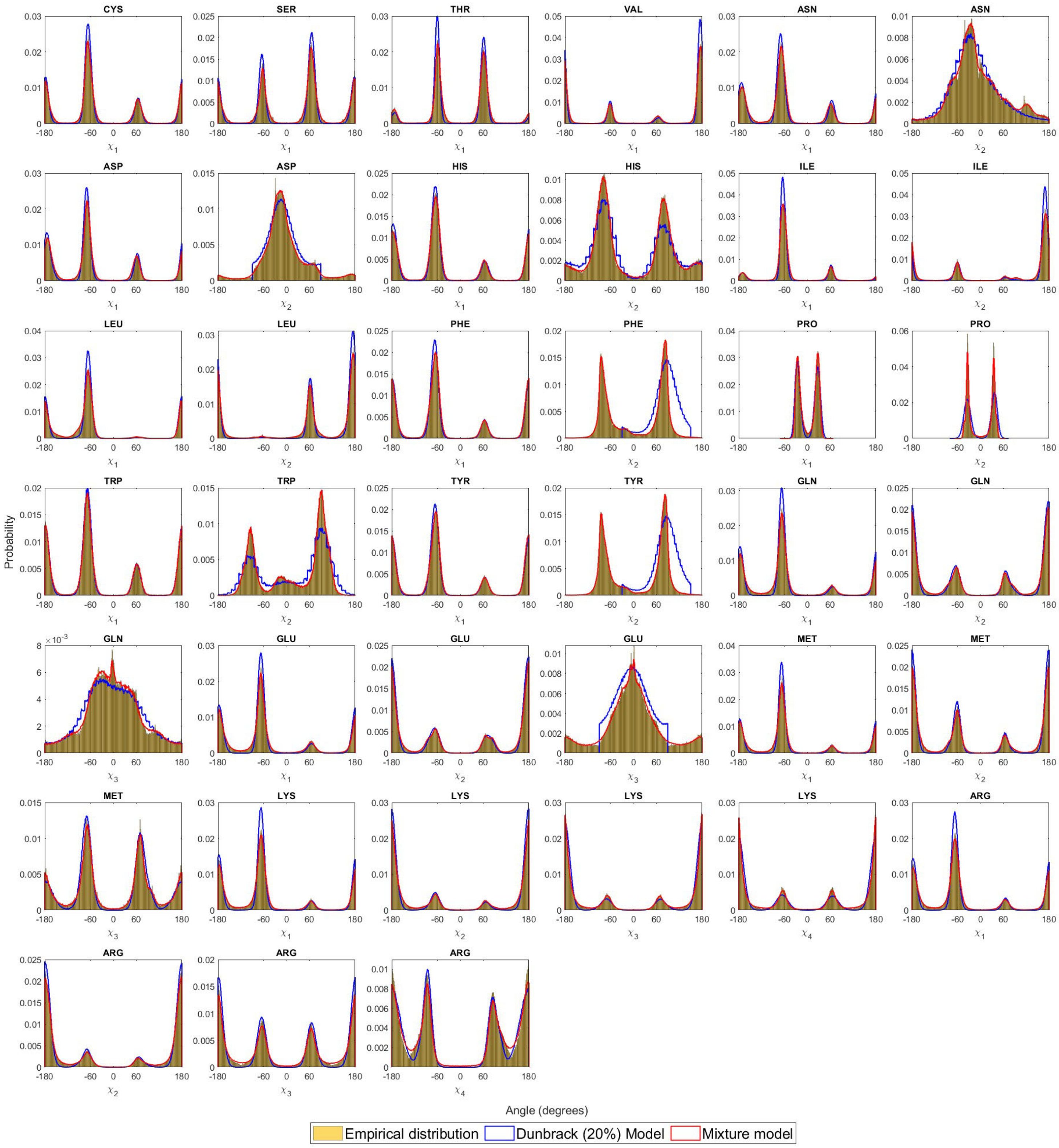

Figure SF 10. Fidelity of the inferred MML mixure models: the projected distribution of individual sidechain dihedral angles across all amino acids derived by randomly sampling N $^{(aa)}$ datapoints (see Table ST 7) from MML derived mixture models and Dunbrack (20% smoothed) library, and compared to the empirical distribution.

S12.8 Model fit across all amino acid sidechain dihedral angles for PDB50 dataset: Qualitative comparison for 25% smoothing level of Dunbrack library

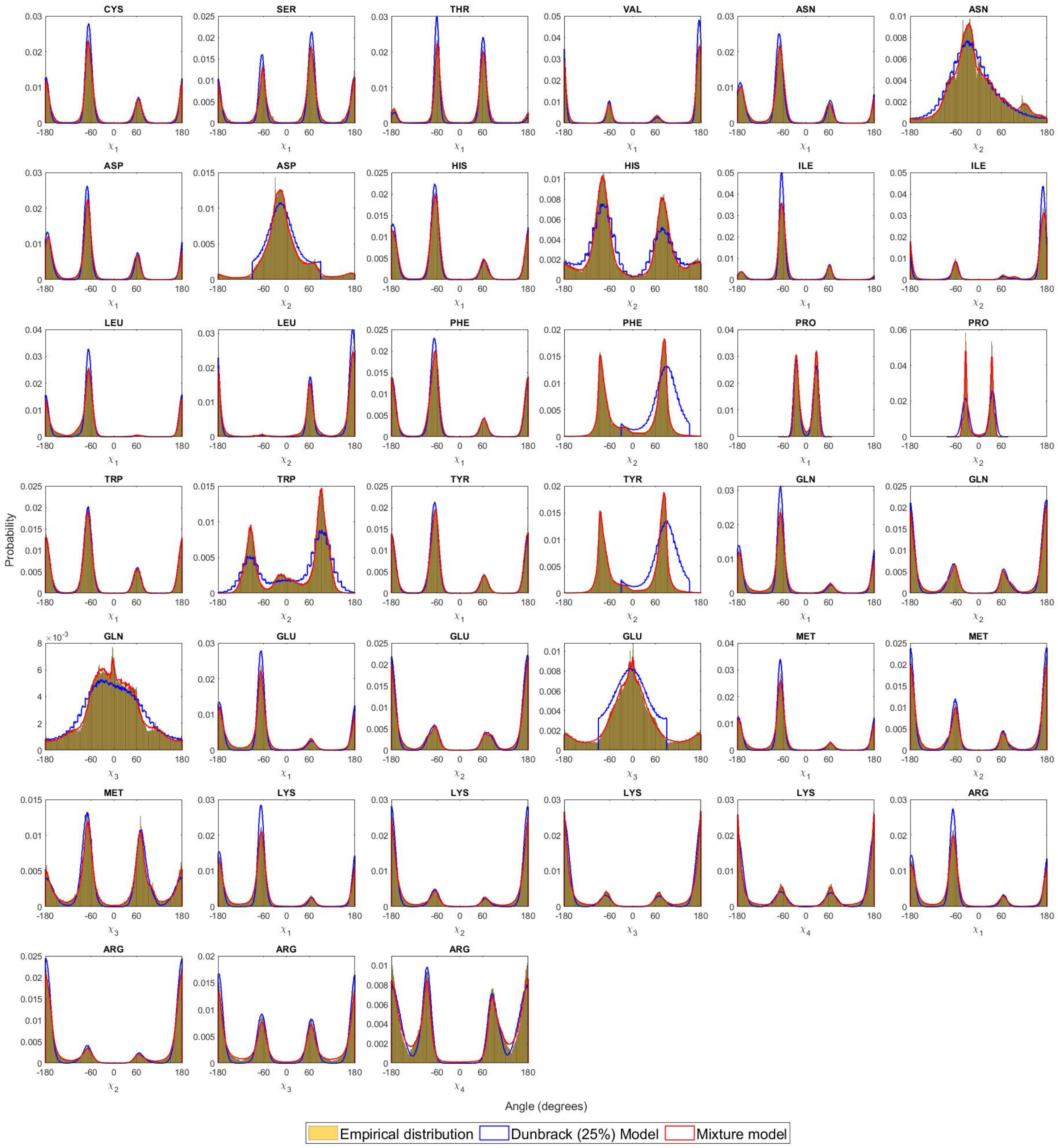

Figure SF 11. Fidelity of the inferred MML mixure models: the projected distribution of individual sidechain dihedral angles across all amino acids derived by randomly sampling $N^{(aa)}$ datapoints (see Table ST 8) from MML derived mixture models and Dunbrack (25% smoothed) library, and compared to the empirical distribution.

S13 Testing Mixture models for overfitting

The MML framework provides a trade-off between model complexity and model fit when inferring a model (Allison, 2018). In order to assess the MML-derived mixture models' ability to capture the underlying dihedral angle distribution without overfitting to the data. We conducted a test to access the fidelity of the derived mixture models to explain an unforeseen dataset. For this test, we considered a collection of 2,238 protein structures that is part of the 2010 Dunbrack Rotamer Library but was not included in our dataset. We compared the dihedral angles calculated from these structures against data sampled from mixture models. Figure SF 12 illustrate how well mixture models capture the underlying distribution, even when the empirical distribution comprises dihedral angles that were not a part of the initial training set. Additionally, Dunbrack's model (with 5%) is included in the same figure for further comparison.

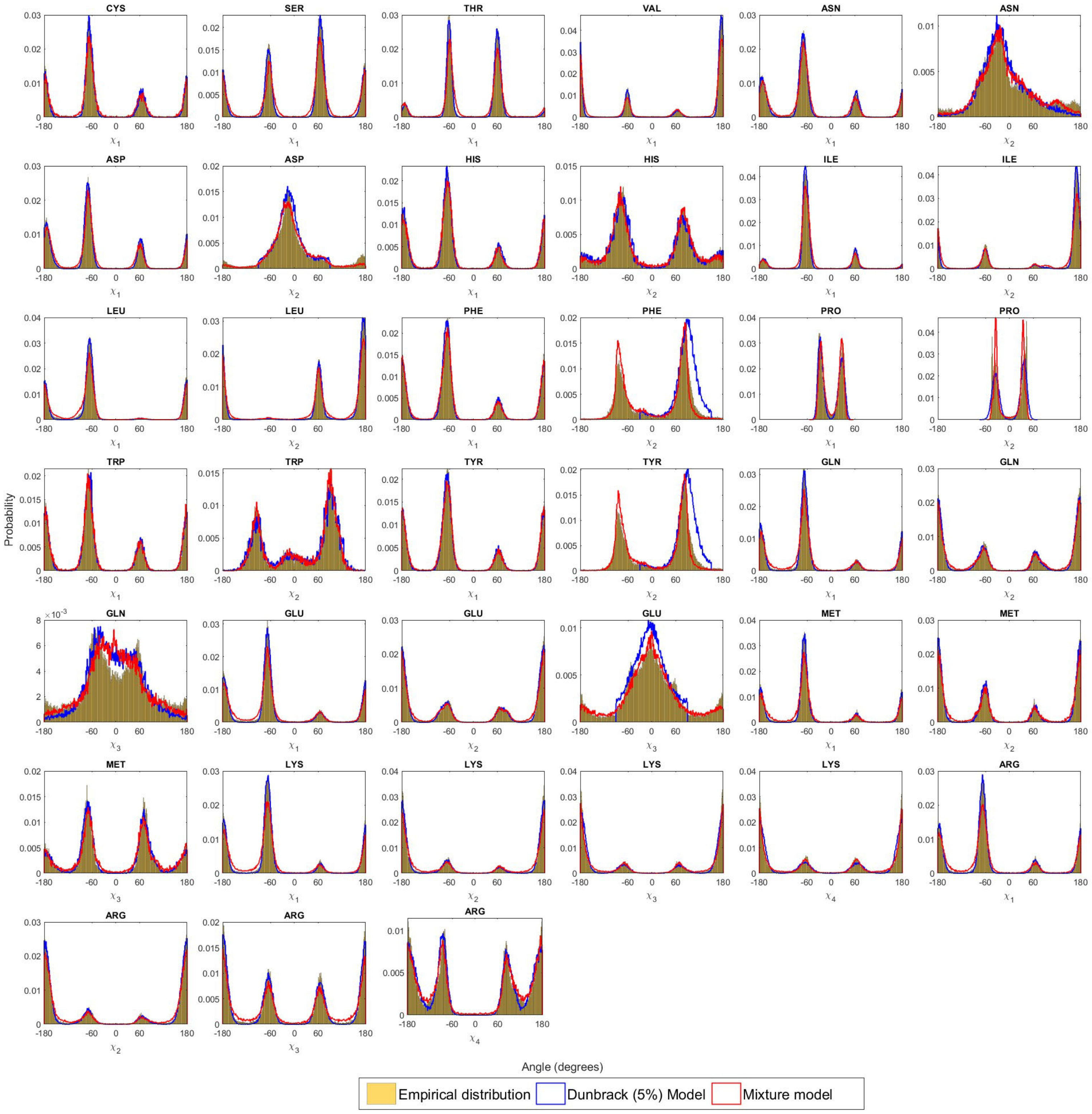

Figure SF 12. the projected distribution of individual sidechain dihedral angles across all amino acids derived by randomly sampling data points from MML-derived mixture models compared against an empirical dataset that is not a part of the mixture models training set.

S14 Assessing the validity of the PDB50HighRes dataset

We assessed the quality of the PDB50HighRes dataset curated from the Protein Data Bank with the corresponding refined protein structures from the PDB-REDO databank (van Beusekom *et al.*, 2018). For each amino acid, a collection of d-dimensional deviation (δ) vectors was created by calculating the deviation of each dihedral angle of the corresponding residue in the PDB-REDO refined structure and in the PDB50HighRes structure. The resulting collection of δ dihedral angle vectors was then statistically analyzed to assess the validity of the dataset used for this research work. The mean and standard deviations of the deviations are presented in Table ST 9, demonstrating that the differences are insignificant.

Table ST 9. This table illustrates mean and standard deviation (SD) statistics (in radians) of dihedral angle deviations δ between PDB-REDO refined structures for the PDB50HighRes dataset. Statistics are presented per each amino acid (a.a) and per each dihedral angle $\langle \phi, \psi, \chi_1, \chi_2, ... \rangle$.

	ϕ_δ (rad)		ψ_δ (rad)		$\chi{1}_\delta$ (rad)		$\chi{2}_\delta$ (rad)		$\chi{3}_\delta$ (rad)		$\chi{4}_\delta$ (rad)		$\chi{5}_\delta$ (rad)	
a.a	Mean	SD	Mean	SD	Mean	SD	Mean	SD	Mean	SD	Mean	SD	Mean	SD
ALA	-0.00650	0.05218	0.00590	0.04708										
GLY	-0.00029	0.06921	-0.00162	0.06539										
VAL	-0.00301	0.04698	0.00500	0.03940	-0.00145	0.04974								
SER	-0.00808	0.05958	0.00670	0.05394	-0.00486	0.10115								
THR	-0.00615	0.05375	0.00673	0.04564	-0.00734	0.05934								
CYS	-0.00581	0.04966	0.00694	0.04315	-0.00590	0.07286								
ILE	-0.00424	0.04689	0.00581	0.03853	-0.00568	0.04758	0.00321	0.09040						
LEU	-0.00588	0.04780	0.00600	0.04109	-0.00374	0.05630	0.00193	0.06668						
PRO	-0.00459	0.05462	0.00138	0.05284	-0.00636	0.11185	0.00589	0.16209						
PHE	-0.00505	0.04714	0.00645	0.04240	-0.00499	0.03757	-0.00060	0.05874						
TRP	-0.00525	0.04744	0.00681	0.04000	-0.00516	0.03472	-0.00033	0.04347						
TYR	-0.00613	0.04606	0.00985	0.04041	-0.00469	0.03492	-0.00192	0.05799						
ASP	-0.00431	0.05880	0.00566	0.05419	-0.00507	0.07146	-0.00214	0.13428						
HIS	-0.00618	0.05235	0.00733	0.04772	-0.00348	0.04822	-0.00209	0.09082						
ASN	-0.00330	0.05627	0.00517	0.05292	-0.00461	0.06436	-0.00053	0.11478						
GLU	-0.00764	0.05770	0.00739	0.05310	-0.00261	0.10601	0.00164	0.10872	-0.00180	-0.00180				
MET	-0.00652	0.05297	0.00729	0.04562	-0.00223	0.08656	0.00091	0.08828	-0.00329	-0.00329				
GLN	-0.00708	0.05416	0.00682	0.04951	-0.00363	0.09195	0.00052	0.09428	-0.00124	-0.00124				
LYS	-0.00651	0.05830	0.00642	0.05240	-0.00282	0.10009	0.00096	0.13190	-0.00053	-0.00053	-0.00077	-0.00077		
ARG	-0.00621	0.05386	0.00902	0.04920	-0.00100	0.09482	-0.00085	0.10300	-0.00224	-0.00224	-0.00026	-0.00026	0.00025	0.08824

References

Allison, L. (2018). *Coding Ockham's Razor*. Springer.

Banerjee, A. *et al.* (2005). Clustering on the unit hypersphere using von mises-fisher distributions. *Journal of Machine Learning Research*, **6**(9).

Kasarapu, P. and Allison, L. (2015). Minimum message length estimation of mixtures of multivariate gaussian and von mises-fisher distributions. *Machine Learning*, **100**(2), 333–378.

Kullback, S. and Leibler, R. A. (1951). On information and sufficiency. *The annals of mathematical statistics*, **22**(1), 79–86.

Shapovalov, M. V. and Dunbrack Jr, R. L. (2011). A smoothed backbone-dependent rotamer library for proteins derived from adaptive kernel density estimates and regressions. *Structure*, **19**(6), 844–858.

van Beusekom, B. *et al.* (2018). Homology-based hydrogen bond information improves crystallographic structures in the pdb. *Protein Science*, **27**(3), 798–808.

Wallace, C. S. and Freeman, P. R. (1987). Estimation and inference by compact coding. *Journal of the Royal Statistical Society: Series B (Methodological)*, **49**(3), 240–252.
